# Risk Factors for Severe Pediatric Invasive Group A Streptococcal Disease

**DOI:** 10.1001/jamanetworkopen.2025.27717

**Published:** 2025-08-19

**Authors:** Evelien B. van Kempen, Adam J. Tulling, Erik G. J. von Asmuth, Leontien B. van der Aa, Else M. Bijker, Merijn Bijlsma, Dorine M. Borensztajn, Caroline L. H. Brackel, Brechje de Gier, Marlies A. van Houten, Monique A. M. Jacobs, Marianne Koenraads, Anjali I. G. Kooter, Ankie Lebon, Jan W. M. van der Linden, Lonneke van Onzenoort-Bokken, Rianne Oostenbrink, Joffrey van Prehn, Nina M. van Sorge, Kim Stol, Gerdien. A. Tramper-Stranders, Aline R. Verhage, Kirsten N. G. van de Weijer, Joanne G. Wildenbeest, Navin P. Boeddha, Mirjam van Veen, Emilie P. Buddingh

**Affiliations:** 1 Department of Pediatrics, Juliana Children’s Hospital, Haga Hospital, the Hague, the Netherlands; 2 Department of Pediatrics, Erasmus Medical Center Sophia, Rotterdam, the Netherlands; 3 Department of Paediatrics, Willem-Alexander Children’s Hospital, Leiden University Medical Center, Leiden, the Netherlands; 4 Department of Pediatrics, Zaans Medical Center, Zaandam, the Netherlands; 5 Department of Pediatrics, Maastricht University Medical Centre, MosaKids Children’s Hospital, Maastricht, the Netherlands; 6 Department of Pediatrics, Oxford Vaccine Group, University of Oxford, Oxford, United Kingdom; 7 Department of Pediatrics, Amsterdam University Medical Center, University of Amsterdam, Amsterdam, the Netherlands; 8 Department of Pediatrics, Noordwest Ziekenhuisgroep, Alkmaar and Den Helder, the Netherlands; 9 Department of Pediatrics, Tergooi Medical Center, Hilversum, the Netherlands; 10 Centre for Infectious Disease Control, National Institute for Public Health and the Environment, Bilthoven, the Netherlands; 11 Department of Pediatrics, Spaarne Gasthuis, Haarlem and Hoofddorp, the Netherlands; 12 Department of Pediatrics, Slingeland Ziekenhuis, Doetinchem, the Netherlands; 13 Department of Pediatrics, Haaglanden Medisch Centrum, the Hague, the Netherlands; 14 Department of Pediatrics, Alrijne Ziekenhuis, Leiderdorp, the Netherlands; 15 Department of Pediatrics, Albert Schweitzer Hospital, Dordrecht, the Netherlands; 16 Department of Pediatrics, Bernhoven, Uden, the Netherlands; 17 Department of Pediatrics Máxima Medisch Centrum Veldhoven, the Netherlands; 18 Department of Medical Microbiology and Infection Prevention, Leiden University Center for Infectious Diseases, Leiden University Medical Center, Leiden, the Netherlands; 19 Department of Medical Microbiology and Infection Prevention, Amsterdam University Medical Center, University of Amsterdam, Amsterdam, the Netherlands; 20 Netherlands Reference Laboratory for Bacterial Meningitis, Amsterdam University Medical Center, Amsterdam, the Netherlands; 21 Department of Pediatrics, Radboud University Medical Center, Amalia Children’s hospital, Nijmegen, the Netherlands; 22 Department of Pediatrics, Franciscus Gasthuis & Vlietland, Rotterdam, the Netherlands; 23 Department of Pediatric Infectious Diseases & Immunology, Beatrix Children’s Hospital University Medical Center Groningen, Groningen, the Netherlands; 24 Department of Pediatric Infectious Diseases and Immunology, Wilhelmina Children’s Hospital, University Medical Center Utrecht, Utrecht, the Netherlands; 25 Department of Pediatrics, Maasstad Hospital, Rotterdam, the Netherlands; 26 Department of Pediatrics, Division of Pediatric Infectious Diseases and Immunology, Erasmus MC-Sophia Children’s Hospital, University Medical Center Rotterdam, Rotterdam, the Netherlands

## Abstract

**Importance:**

An increase in pediatric cases of invasive group A streptococcus (iGAS) disease was noted in the Netherlands starting in early 2022. GAS disease can range from mild to life-threatening invasive infections. Clinical and public health decisions rely on timely and detailed reporting of clinical data.

**Objective:**

To determine risk factors associated with severe pediatric iGAS, defined as requiring admission to an intensive care unit and/or death, and to analyze pediatric iGAS incidence, presentations, and outcome between pre–COVID-19 pandemic (January 2015 to March 2020), COVID-19 pandemic (April 2020 to December 2021), and post–COVID-19 pandemic (January 2022 to June 2024) periods.

**Design, Setting, and Participants:**

This observational, retrospective and prospective cohort study in 20 hospitals (tertiary and nontertiary) in the Netherlands was conducted from January 2015 to June 2024, with real-time reporting of data on the study website since January 2022. Children aged 0 to 17 years with iGAS (positive culture or polymerase chain reaction test and/or clinical presentation) were included.

**Exposure:**

iGAS infection.

**Main Outcome and Measures:**

The primary outcome was risk factors for severe iGAS; secondary outcomes included iGAS incidence rate and clinical phenotypes prior, during, and after the COVID-19 pandemic. Risk factors for severity and mortality were analyzed using univariable and multivariable logistic regression analyses, and incidence rate ratios (IRRs) between pre–COVID-19 and postCOVID-19 pandemic periods were calculated using Poisson regression.

**Results:**

Of 617 children included, 351 (56.9%) were aged 0 to 4 years. For the 192 participants with detailed data collection, median (IQR) age was 4.2 (1.7-7.1) years and 91 (47.4%) were male. iGAS cases decreased during the COVID-19 pandemic and increased significantly in the post–COVID-19 period (IRR, 2.93; 95%, 2.46-3.49), as compared with the pre–COVID-19 pandemic period. By late 2023, the incidence of iGAS returned to pre–COVID-19 pandemic levels. Factors associated with increased risk of severe disease included a post–COVID-19 pandemic diagnosis (odds ratio [OR], 3.49; 95% CI, 2.31-6.26), pulmonary involvement (OR, 8.64; 95% CI, 5.50-13.55), streptococcal toxic shock syndrome (STSS; OR, 11.71; 95% CI, 4.39-31.18), and meningitis or encephalitis (OR, 4.38; 95% CI, 4.39-31.18). Clinical factors associated with increased risk of severe disease were reduced consciousness (OR, 7.61; 95% CI, 1.84-34.41), dyspnea (OR, 9.89; 95% CI, 3.04-32.14), abnormal auscultation (OR, 6.32; 95% CI, 2.18-18.32), and elevated C-reactive protein (OR, 6.32; 95% CI, 2.18-18.32), while estimated glomerular filtration rate was associated with a decreased risk (OR, 0.64; 95% CI, 0.49-0.84). Disease severity increased in post–COVID-19-pandemic cases, with higher mortality (13 of 294 cases [4.4%] vs 3 of 218 cases [1.4%]) and intensive care admission rates (113 of 294 cases [38.4%] vs 34 of 218 cases [15.6%]), as compared with pre–COVID-19 pandemic cases. Severity of pulmonary iGAS was similar in both periods. In the post–COVID-19 pandemic period, there was a significant increase in the incidence of pulmonary infections (IRR, 5.04; 95% CI, 3.27-7.97) , STSS (IRR, 10.30; 95% CI, 3.88-35.60), meningitis or encephalitis (IRR, 12.30; 95% CI, 4.14-52.70), and necrotizing fasciitis (IRR, 26.10; 95% CI, 5.14-475.00).

**Conclusions and Relevance:**

In this cohort study, risk factors for a complicated course of iGAS in children included pulmonary or central nervous system involvement, STSS, reduced consciousness or pulmonary clinical signs, elevated CRP, and decreased eGFR. Awareness of these risk factors is important to improve timely recognition of at-risk cases and improve clinical outcomes.

## Key Points

**Question:**

What are the risk factors associated with severe invasive group A streptococcus (iGAS) disease, defined a**s** intensive care admission and/or death?

**Findings:**

In this cohort study of 617 children with pediatric iGAS, severe disease was associated with a post–COVID-19 pandemic diagnosis, pulmonary involvement, streptococcal toxic shock syndrome, and meningitis or encephalitis. Significant clinical risk factors were reduced consciousness, dyspnea, abnormal auscultation, elevated C-reactive protein, and decreased estimated glomerular filtration rate.

**Meaning:**

The identification of specific risk factors for severe iGAS may raise awareness among clinicians to recognize at-risk cases and to improve clinical outcomes.

## Teaser

This cohort study analyzes factors associated with severe invasive group A streptococcus infection before, during, and after the COVID-19 pandemic among children in the Netherlands.

## Introduction

After the COVID-19 pandemic, several countries, including the Netherlands, reported a surge of invasive group A streptococcus (iGAS) infections.^1,2,3,4,5,6,7,8,9^ *Streptococcus pyogenes* (GAS) spreads via droplets, direct or indirect contact (ie, secretions and fomites), and possibly airborne transmission.^10^ Approximately 1% to 5% of healthy adults and 10% of school-aged children are asymptomatic carriers of GAS in the nasopharynx or on the skin.^11,12,13^ GAS causes a wide range of infections, varying from mild noninvasive illnesses, like pharyngitis, to severe, potentially fatal invasive infections, like meningitis and sepsis. Seasonal iGAS surges are common, and a slight increase in iGAS incidence was observed in some countries prior to the emergence of COVID-19.^14,15^ During the COVID-19 pandemic, iGAS cases dropped sharply, presumably due to nonpharmaceutical interventions, like wearing face masks and lockdowns, aimed at reducing SARS-CoV-2 transmission.^1,3,8,16^

In 2022, a Dutch survey showed a 2-fold increase in pediatric iGAS cases between July 2021 and June 2022 as compared with pre–COVID-19 years (2018-2019).^1^ This increase was accompanied by a shift in clinical presentations, with an increased incidence of pneumonia with empyema and necrotizing fasciitis (NF). Similarly, the Dutch National Institute for Public Health and the Environment reported a 7-fold increase in annual notifications of NF and streptococcal toxic shock syndrome (STSS) in children aged 0 to 5 years in 2022 vs 2016 to 2019.^17^ However, these reports lacked systematical case recording and detailed information on clinical characteristics, treatment, or outcome. Therefore, we initiated a multicenter, nationwide cohort study. From 2022 onwards, data were reported daily on our online real-time dashboard^18^ to provide health professionals, public health policymakers, and the general public with up-to-date information on the trend, severity, and clinical characteristics of pediatric iGAS in the Netherlands. Our primary aim was to determine risk factors for severe pediatric iGAS, defined as pediatric intensive care unit (PICU) admission and/or death. Secondary aims were to analyze pediatric iGAS incidence, presentations, and outcome between pre–COVID-19 pandemic (January 2015 to March 2020), COVID-19 pandemic (April 2020 to December 2021), and post–COVID-19 pandemic (January 2022 to May 2024) periods.

## Methods

### Study Design, Setting, and Participants

This national, multicenter cohort study was evaluated by the medical ethical review committee of Leiden University Medical Center. The study was conducted in 20 hospitals, including all 7 Dutch academic hospitals with PICUs (5 with full inclusions and 2 with partial inclusions) (eFigure 1 in Supplement 1). Participating hospitals were part of the nation-wide Clinical Features of COVID-19 in Pediatric Patients (COPP) consortium, which previously studied pediatric COVID-19 and multisystem inflammatory syndrome in children.^19^ Study periods were defined as: pre–COVID-19 pandemic period (January 2015 to March 2020), COVID-19 pandemic period (April 2020 to December 2021), and post–COVID-19 pandemic period (January 2022 [because relaxation of COVID-19 restrictions after hard lockdown started then] to May 2024).^20^ Patients were included prospectively between January 2022 and June 2024, and retrospectively from January 2015 to June 2024. Informed consent was obtained from parents or caregivers and children older than 12 years, if applicable. Written informed consent was obtained for the prospective cases with elaborate data collection. Complying with article 7:458 of the Civil Code (BW), the medical ethical review committee waived informed consent for retrospective collection of concise data. Study protocols are available on the study website.^18^ The Strengthening the Reporting of Observational Studies in Epidemiology (STROBE) reporting guideline was followed.

Inclusion criteria were children aged 0 to 17 years with an in-hospital diagnosis of iGAS (emergency department presentation, hospitalization, or postmortem, including deaths before hospital arrival). iGAS was defined as (1) positive culture or molecular detection (polymerase chain reaction–based methods) of GAS in a normally sterile body site such as blood, cerebrospinal fluid, pleural fluid, or synovial fluid; (2) clinical diagnosis of STSS or NF (with or without microbiological confirmation); or (3) positive molecular detection or culture of GAS from a nonsterile body site with clinical features compatible with iGAS with no other causative microorganism isolated (list of sterile sites in eTable 1 in Supplement 1). Patients were retrospectively identified through the hospital’s local microbiological databases, and/or prospectively by local investigators.

### Data Collection

Data were retrieved from electronic health records. Cases from 2022 onwards were prospectively included with informed consent. Detailed data collection in these cases included medical history, health care encounters in the 14 days preceding iGAS hospitalization, exposure to iGAS, admission duration, laboratory results, treatments, and interventions. Ethnicity data were not available. For cases without informed consent (diagnosed between 2015 and 2022 or after 2022 but who could not be traced), concise data were collected under a waiver. These data included: age group (younger than 5 years, 5-9 years, and 10 years and older), month and year of iGAS diagnosis, precoinciding and/or coinciding infections, diagnostic category of iGAS, and outcome. Since April 2022, Dutch medical microbiologist laboratories were requested to submit GAS isolates from sterile sites to the National Reference Laboratory for Bacterial Meningitis (NRLBM) for genotyping. The *emm*-type data were collected from electronic health records of the patient or linked through probabilistic linkage to the data obtained from the NRBLM database for prospectively included patients. Data were collected using Castor Electronic Data Capture. We developed an online dashboard for real-time trend monitoring and analysis.^18^ Reporting and analyses were automated using an R script.^21^ Data handling for this study complies with General Data Protection Regulation 2016/679.

### Outcome Measures

The primary outcome measure was risk factors for severe iGAS, defined as PICU admission and/or death. Secondary outcome measures were risk factors for iGAS-related mortality and iGAS incidence rate and clinical phenotypes prior, during, and after the COVID-19 pandemic.

Children with multiple clinical diagnoses (eg, pneumonia and sepsis) were allocated to clinical groups based on a sequential decision-making process, utilizing prespecified clinical syndromes at presentation or during admission (eFigure 2 in Supplement 1). Priority was given to localizing clinical syndromes (eg, children with pulmonary infection and sepsis were allocated to the pulmonary infection group). Cases not fitting any category were allocated to the other category.

### Statistical Analysis

Descriptive values are reported in absolute numbers with percentages for categorical values or median and IQRs for continuous values. Analyses were performed using R software version 4.2.1 (R Project for Statistical Computing). iGAS incidence rate ratios (IRRs) between the periods before and after the COVID-19 pandemic were calculated using a Poisson regression model corrected for period length. Risk factors for severity and mortality were analyzed using univariable logistic regression analyses with confidence intervals estimated using the sandwich estimator (sandwich R package). For variables exhibiting (near) complete separation, the Firth penalized likelihood method was applied, which provides its own corrected confidence intervals and thus did not require sandwich-based adjustment.

Missing values (limited to prodromal signs) were imputed using the predictive mean matching algorithm (mice R package). Multivariable analyses were done with variable selection using the backward and forward selection method with multivariable logistic regression based on the bayesian information criterion.

In patients with detailed clinical and laboratory data available (including *emm*-type), we evaluated associations of clinical or laboratory parameters with severity, adjusting for clinical syndrome group (eg, pulmonary infection or soft-tissue infection), age, and sex. Fisher exact tests and χ^2^ tests were used for contingency analyses, and the Kruskal-Wallis rank sum test was used for comparisons of continuous data. A Benjamini-Hochberg–corrected *P* < .05 was considered significant.

## Results

### General Cohort Characteristics

**Figure 1.**
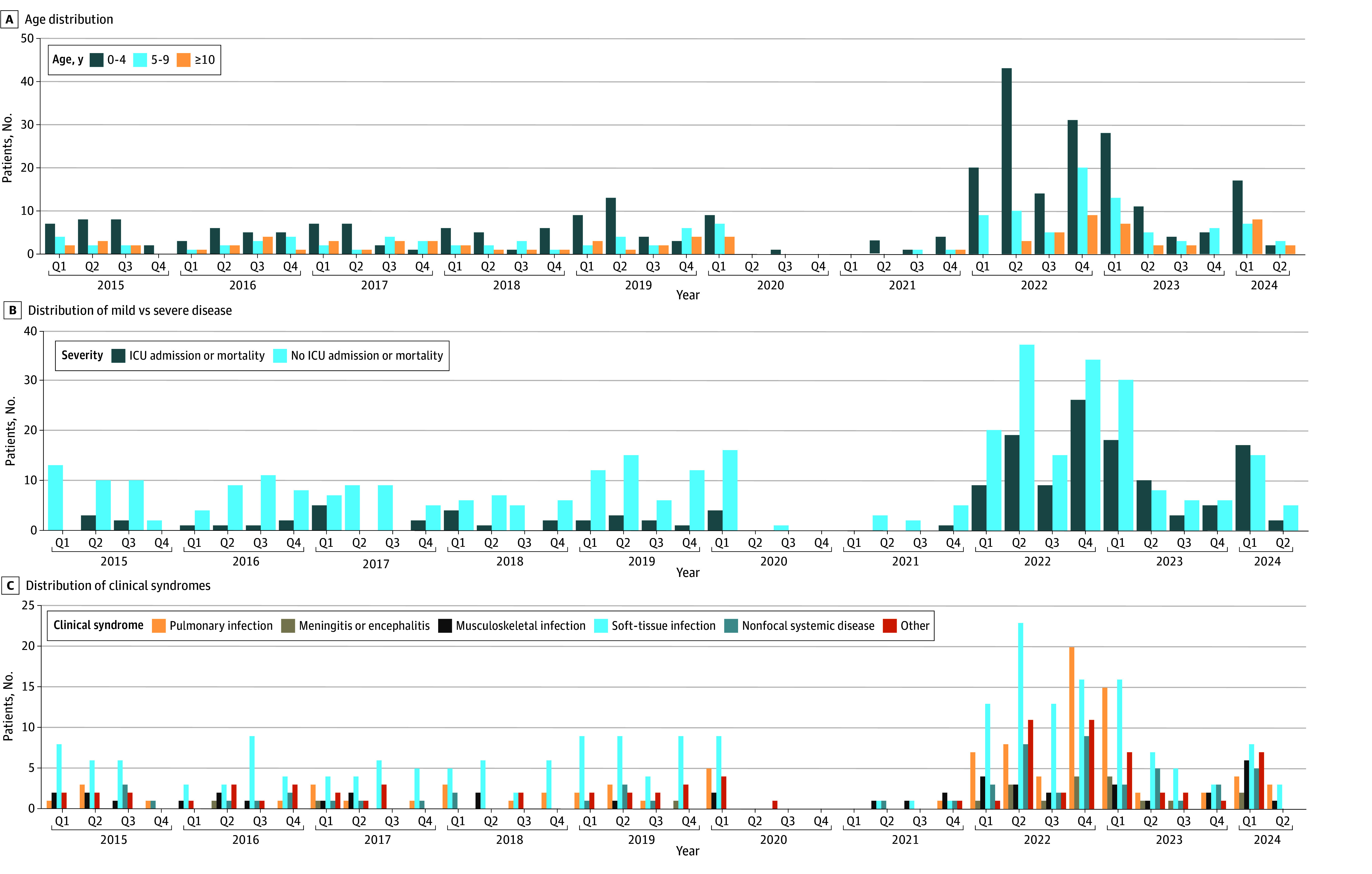
Summary of Clinical Characteristics Epidemiological curves of inclusions (age [A], mild vs severe disease [B], and clinical syndromes [C]) in the study period in hospitals participating in the retrospective as well as in the prospective part of the study. Severe disease was defined as intensive care unit (ICU) admission or death. Q indicates quarter.

We included 617 children aged 0 to 17 years with iGAS between January 2015 and June 2024 (Table 1). For the 192 participants with detailed data collection, median (IQR) age was 4.2 (1.7-7.1) years, 91 (47.4%) were male, and 90 (46.9%) had a prior medical history, most commonly recurrent ear, nose, and throat infections (27 of 192 children [14.0%]). Among these 27 children, 3 had an underlying condition possibly predisposing them to a more severe course of iGAS (Down syndrome in 2 children and Kabuki syndrome in 1 child), 5 had mild pulmonary comorbidities (eg, asthma), and the remainder had no relevant comorbidity. Of the 192 patients, 21 (11.0%) had prior pulmonary conditions such as asthma (7 of 192 children [3.6%]), and 15 patients (7.8%) had dermatological conditions, most commonly eczema (12 of 192 children [ 6.2%]). None had a known immunodeficiency (eTable 2 in Supplement 1). Of all 617 cases, 218 (35.3%) occurred during the pre–COVID-19 period (January 2015 to March 2020), 12 (1.9%) occurred during the COVID-19 pandemic (April 2020 to December 2021), and 387 (62.7%) occurred in the post–COVID-19 period (January 2022 to May 2024). Most children were younger than 5 years (351 of 617 children [56.9%]) (Table 1 and Figure 1 A-C). Severe disease occurred in 195 of 617 cases (31.6%); of these 195 severe cases, there were 182 PICU admissions (93.3%) and 28 deaths (14.4%) (Table 1). In deceased children, the top 3 clinical presentations were nonfocal systemic disease (10 children [35.7%]), pulmonary infection (5 children [17.9%]), and meningitis or encephalitis (4 children [14.3%]).

**Table 1.** Clinical Patient Characteristics of Children With Invasive Group A Streptococcal Disease Infection^a^

Characteristic	Participants, No. (%) (N = 617)
Overall data collection	
Age group, y	
0-4	351 (56.9)
5-9	166 (26.9)
10-17	100 (16.2)
COVID-19 pandemic periods	
January 2015 to March 2020	218 (35.3)
April 2020 to December 2021	12 (1.9)
January 2022 to May 2024	387 (62.7)
Severe disease	
Total	195 (31.6)
Intensive care unit admission	182 (29.5)
Mortality	28 (4.5)
Clinical group^b^	
Pulmonary infection	118 (19.1)
Meningitis or encephalitis	26 (4.2)
Necrotizing fasciitis	16 (2.6)
Musculoskeletal infection	63 (10.2)
Soft tissue infection (non–necrotizing fasciitis)	226 (36.6)
Nonfocal systemic disease	66 (10.7)
Other	102 (16.5)
Detailed data collection^c^	
Participants, No./total No. (%)	192/617 (31.1)
Sex, No./total No. (%)	
Female	101/192 (52.6)
Male	91/192 (47.4)
Age, median (IQR), y	4.2 (1.7-7.1)
Medical history present, No./total No. (%)	90/192 (46.9)

^a^ Admitted to hospital between January 2015 and May 2024 due to invasive group A streptococcal disease infection.

^b^ Categorized (1 option per patient).

^c^ For a subset of patients, more detailed clinical data were collected.

Soft-tissue infections (excluding NF) accounted for 226 of 617 clinical presentations (36.6%), followed by pulmonary infections in 118 children (19.1%) and nonfocal systemic diseases in 66 children (10.7%). Among pulmonary infection cases, 86 children (72.9%) had empyema and 31 (26.3%) had bacteremia (21 of 86 children [24.4%] with and 10 of 32 children [31.3%] without empyema). Of 26 cases with central nervous system infection (ie, meningitis or encephalitis), 9 (34.6%) had concurrent sepsis. Clinical presentations did not differ significantly between age groups (eTable 3 in Supplement 1). Overlap between clinical syndromes and clinical groups is shown in eFigure 2 in Supplement 1.

A preceding airway infection and/or flu-like symptoms was present in 201 of 572 cases (35.1%) and in 65 of 113 cases (57.5%) with pulmonary iGAS. A prior skin condition was present in 194 of 573 cases (33.9%) (eg, varicella zoster virus [VZV] infection or eczema) (eTable 3 in Supplement 1). VZV infection preceded NF in 12 of 16 cases (75.0%), significantly more often than in other clinical groups (73 of 554 cases [13.2%; 95% CI, 1.2%-17.0%]; *P* < .001). Medical history did not differ significantly between clinical presentations of iGAS, except for ear, nose, and throat conditions, which were more often present in meningitis or encephalitis cases or cases included in the other disease category (eTable 2 in Supplement 1). For 192 of 617 post–COVID-19 pandemic cases (31.1%), detailed clinical and laboratory information was available.

### Risk Factors for Severe Disease

**Figure 2.**
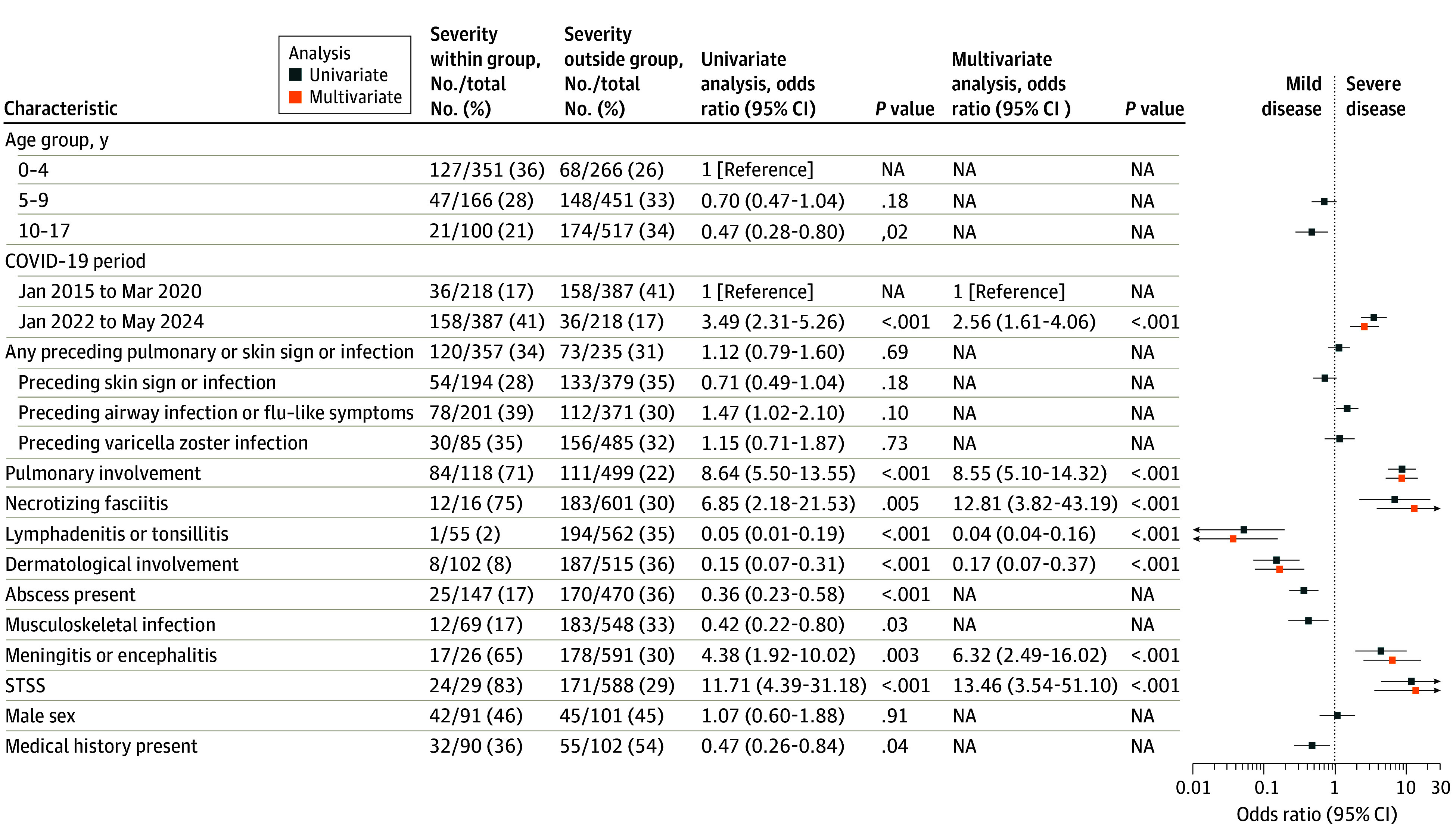
Risk Factors for Severe Disease in Children With Invasive Group A Streptococcal Disease Infection Severe disease was defined as intensive admission and/or mortality. Log odds ratios were calculated based on a univariable and multivariable logistic regression models. The multivariable model did not account for a prior medical history due to the fact this variable was only collected in case of obtainment of informed consent. NA indicates not applicable; STSS, streptococcal toxic shock syndrome.

In a univariable analysis, presentation in the post–COVID-19 pandemic period (odds ratio [OR], 3.49; 95% CI, 2.31-6.26), pulmonary involvement (OR, 8.64; 95% CI, 5.50-13.55), NF (OR, 6.85; 95% CI, 2.18-21.53), STSS (OR, 11.71; 95% CI, 4.39-31.18), and meningitis or encephalitis (OR, 4.38; 95% CI, 4.39-31.18) were significantly associated with increased risk of severe disease (PICU admission and/or death) (Figure 2). Children younger than 5 years had more severe disease than children aged 10 to 17 years. STSS was associated with mortality regardless of PICU admission (eFigure 3 in Supplement 1).

In the multivariable analysis, most risk factors were independently and significantly associated with severity, except for age, which did not improve the fitness of the model. Preceding infections, including VZV infection, were not associated with severity (Figure 2). Severity of pulmonary iGAS was similar between the pre–COVID-19 and post–COVID-19 pandemic periods, but in nonpulmonary cases, presentation in the post–COVID-19 pandemic period was highly associated with more severe disease (OR, 3.44; 95% CI, 1.94-6.38; *P* < .001) (eFigure 4 and eFigure 5 in Supplement 1). In patients with availability of detailed data, multivariable analysis adjusted for age, sex, and clinical group revealed reduced consciousness (OR, 7.61; 95% CI, 1.84-34.41), dyspnea (OR, 9.89; 95% CI, 3.04-32.14), abnormal findings on auscultation (OR, 6.32; 95% CI, 2.18-18.32), and elevated C-reactive protein (OR, 1.62; 95% CI, 1.10-2.39) were associated with increased risk of severe disease, while estimated glomerular filtration rate was associated with decreased risk of severe disease (OR, 0.64; 95% CI, 0.49-0.84) (eFigure 6 and eFigure 7 in Supplement 1).

### GAS *emm*-Type

GAS was most frequently detected in blood (69 of 177 children [39.0%]) and pleural fluid (39 of 177 children [22.0%]), primarily via culture (162 of 176 children [92.0%]), and in 16 of 176 cases (9.1%), with polymerase chain reaction (eTable 4 in Supplement 1). The *emm*-type could be inferred for 101 of 192 children (52.6%) in the post–COVID-19 pandemic period. The most prevalent *emm*-types were 1.0 (41 of 101 children [40.6%]), 3.93 (20 of 101 children [19.8%]), and 4.0 (11 of 101 children [10.9%]) (eTable 5 in Supplement 1). In the 2022 to 2023 surge, e*mm*-type 1.0 and 12.0 were predominant in 46 of 77 cases (59.7%), but decreased to 2 of 24 cases (8.3%) in 2024, when *emm*-type 3.93 dominated in 17 of 24 cases (70.8%) (eFigure 8 and eTable 5 in Supplement 1).

The *emm-*type 1.0 was detected in 48.1% of severe cases (25 of 52 cases) and 32.6% of mild cases (16 of 49 cases; χ^2^_1_ = 2.49; *P* = .23) (eTable 6 in Supplement 1). However, multivariable analysis revealed that *emm-*types 1.0 and 4.0 were significantly associated with severe iGAS after adjusting for clinical syndrome (eTable 7 in Supplement 1). The *emm*-type 4.0, identified in 11 of 101 cases (10.9%), caused no pulmonary manifestations but accounted for 2 of 5 meningitis or encephalitis cases (40.0%) and 5 of 16 nonfocal systemic disease cases (31.3%) (eFigure 8 and eTable 8 in Supplement 1).

### Differences Before and After the COVID-19 Pandemic

The IRR of iGAS increased significantly in the post–COVID-19 period (IRR, 2.93; 95% CI, 2.46-3.49) (Figure 1A-C and eTable 9 in Supplement 1). The IRR of all types of iGAS infection increased, most notably central nervous system infections (IRR, 12.31; 95% CI, 4.14-52.73), NF (IRR, 26.07; 95% CI, 5.14-474.96), STSS (IRR, 10.32; 95% CI, 3.88-35.59), pulmonary infections (IRR, 5.04; 95% CI, 3.27-7.97). (eTable 9 in Supplement 1). iGAS infections were more severe in the post–COVID-19 pandemic period, with PICU admissions rising from 34 of 218 cases (15.6%) between 2015 and 2020 to 113 of 294 cases (38.4%) in 2022 to 2024 (χ^2^_1_ = 31.90; *P* < .001), and mortality increasing from 3 of 218 cases (1.4%) to 13 of 294 cases (4.4%; χ^2^_1_ = 3.84; *P* = .12). Age distribution was similar between the 2 time periods. All clinical groups showed increased IRRs in the post–COVID-19 period (eTable 9 in Supplement 1). Preceding airway infections were more common in the post–COVID-19 period (119 of 285 cases [41.8%] in 2022-2024 vs 57 of 185 cases [30.8%] in 2015-2020), although this difference was not significant after multiple testing correction (χ^2^_1_ = 5.72; *P* = .05) (Table 2). There was no significant difference in the occurrence of preceding skin problems and/or VZV infections between the 2 periods.

**Table 2.** Clinical Patient Characteristics in Children With Invasive Group A Streptococcal Disease Infection During Pre–COVID-19 and Post–COVID-19 Periods^a^

Characteristic	Participants, No. (%)		*P* value^b^
	Pre–COVID-19: January 2015 to March 2020 (n = 218)	Post–COVID-19: January 2022 to May 2024 (n = 294)	
Age, y			
0-4	117 (53.7)	175 (59.5)	.18
5-9	57 (26.1)	81 (27.6)	
10-17	44 (20.2)	38 (12.9)	
Severe disease			
Intensive care unit admission	34 (15.6)	113 (38.4)	<.001
Mortality	3 (1.4)	13 (4.4)	.12
Clinical group^c^			
Pulmonary infection	28 (12.8)	65 (22.1)	<.001
Meningitis or encephalitis	3 (1.4)	17 (5.8)	
Necrotizing fasciitis	1 (0.5)	12 (4.1)	
Musculoskeletal infection	19 (8.7)	24 (8.2)	
Soft tissue infection (non–necrotizing fasciitis)	114 (52.3)	93 (31.6)	
Non focal systemic disease	18 (8.3)	39 (13.3)	
Other	35 (16.1)	44 (15.0)	
Preceding skin or pulmonary signs, No./total No. (%)^d^			
Either	114/200 (57.0)	201/288 (69.8)	.02
Preceding skin signs	69/193 (35.8)	101/279 (36.2)	.98
Preceding pulmonary signs	57/185 (30.8)	119/285 (41.8)	.05
Preceding varicella zoster infection	30/192 (15.6)	45/277 (16.2)	.93
Clinical involvement			
Pulmonary involvement	28 (12.8)	65 (22.1)	.03
Necrotizing fasciitis	1 (0.5)	12 (4.1)	.04
Systemic involvement (sepsis, STSS, or bacteremia)	36 (16.5)	81 (27.6)	.02
Lymphadenitis or tonsillitis	28 (12.8)	20 (6.8)	.06
Dermatological involvement	36 (16.5)	53 (18.0)	.81
Abscess present	78 (35.8)	56 (19.0)	<.001
Musculoskeletal infection	19 (8.7)	28 (9.5)	.86
Meningitis or encephalitis	3 (1.4)	17 (5.8)	.04

Abbreviation: STSS, streptococcal toxic shock syndrome.

^a^ Only patients from participating centers that included both pre–COVID-19 and post–COVID-19 data were included.

^b^ Statistical testing was performed using the χ^2^ test. *P* value correction was conducted following the Benjamini-Hochberg procedure.

^c^ Categorized (1 option per patient).

^d^ Data on prodromal signs was not available for all patients.

## Discussion

This cohort study found that a marked increase in pediatric iGAS cases occurred in the Netherlands in the post–COVID-19 pandemic period, similar to reports from several other European countries, Australia, and the US.^1,2,3,4,5,6,7,8,9,17,22^ This followed a very large decline in iGAS cases during the COVID-19 pandemic, supporting the hypothesis that nonpharmaceutical interventions aimed at limiting transmission of SARS-CoV-2 also reduced GAS transmission.^1,8,16^ Perhaps the lack of exposure to GAS during the COVID-19 pandemic prevented the development of GAS-specific immunity in children (ie, immunity debt).^23,24^ Additionally, the post–COVID-19 pandemic resurgence of viral infections may have increased susceptibility to secondary GAS infections. These factors may, at least partially, explain the international post–COVID-19 pandemic surge.^25^ It remains unclear whether these factors contribute to the increased severity because severity varied by country. Risk factors for severe iGAS and differences between pre–COVID-19 and post–COVID-19 pandemic iGAS cases remain poorly understood.

We confirmed that children with pulmonary infections, NF, STSS, or central nervous system infections due to iGAS had worse outcomes.^26^ Post–COVID-19 pandemic cases were more severe than pre–COVID-19 pandemic cases, with increased PICU admissions and deaths not explained by a difference in age, but more likely by a shift toward more cases with pulmonary, systemic, or central nervous system infections. Clinical risk factors for PICU-admission or death in our study included reduced consciousness, dyspnea, abnormal auscultation, elevated C-reactive protein, and decreased estimated glomerular filtration rate. Similar to other European countries, we saw a marked increase in pulmonary iGAS cases in the post–COVID-19 pandemic period^19,27^; this was associated with a higher proportion of patients with preceding respiratory tract infections (31% pre–COVID-19 vs 42% post–COVID-19). Because VZV vaccination is not part of the Dutch national immunization program, we evaluated preceding VZV infections. In our post–COVID-19 pandemic cohort, the proportion of children with preceding skin infections such as VZV was similar to pre–COVID-19 pandemic cases (16% pre– and post–COVID-19), similar to Denmark.^8^ In line with this finding, de Gier et al^28,29^ showed that between January 2022 and March 2023, 34% of GAS skin and soft tissue infections were attributable to VZV infections, lower than prior to the COVID-19 pandemic, despite a surge in VZV cases in 2022. Apparently, the interaction between VZV infections and the risk of iGAS appears more complex than previously assumed.^30,31^ Given the role of VZV as a risk factor for severe iGAS, the unchanged proportion of preceding VZV cases does not fully explain the increased severity of post–COVID-19 iGAS.

In addition to host factors (such as reduced immunity to GAS) and the resurgence of viral infections as contributors to increased incidence and severity of iGAS, there may have been a shift to more virulent GAS strains in the post–COVID-19 pandemic period. We found *emm*-types 1.0, 3.93, and 4.0 to be the main contributors to infections in the post–COVID-19 pandemic period, with *emm-*types 1.0 and 4.0 associated with severe disease. This finding aligns with De Gier et al,^17^ who showed that in 2022, multiple *emm-*types were responsible for the surge in Dutch children aged 0 to 5 years, with *emm-*types 1, 4, 12, 22, and 89 accoutning for more than 80% of the isolates. Because national-level bacteriological iGAS surveillance only started in 2019, we were unable to compare current circulating *emm*-types with pre–COVID-19 cases. However, a previous temporal analysis showed that the Dutch surge in iGAS coincided with a significant increase of invasive *emm-*type 1.0 isolates, where the M1_UK_ variant became dominant among invasive *emm*-type 1.0 isolates.^32,33^ A new *emm-*type 4 lineage, M4_NL22_, has also been associated with the iGAS surge in the Netherlands.^34^ In our study, *emm-*type 4.0 was predominant in nonfocal infections, similar to findings from Nygaard et al^8^ and Villalón et al.^35^ We saw a dramatic increase in iGAS caused by *emm-*type 3.93 at the beginning of 2024. Although a previous study implicated this strain to be associated with respiratory and central nervous system infections, there was no association of infections by this *emm*-type with specific clinical syndromes in our cohort.^36^

Although several countries have reported shifts in dominant *emm* types, they differed by country.^6,8,37,38^ Perhaps this is due to differences in the population immunity and strain-associated virulence factors.

A better understanding of the risk factors for severe iGAS in children will enable timely action in case of a new outbreak. In our study, one-third of children seen in hospital with iGAS had severe disease, defined as PICU admission and/or death, with 28 children in our cohort dying due to iGAS. Awareness and prompt recognition of risk factors for severe disease is important to improve clinical outcomes. Our near real-time reporting on the study website has aided in clinical surveillance of pediatric iGAS because the national surveillance system and microbiological surveillance lacked clinical detail. Our study shows that measures to reduce the spread of one pathogen (in this case, nonpharmaceutical interventions to reduce the spread of SARS-CoV-2), can have unwanted effects on the emergence or reemergence of other pathogens as those measures are lifted (as shown in this study, the increase in iGAS IRR and severity).

### Strength and Limitations

This study has many strengths. Our study used uniformly collected data, in one of the largest pediatric cohorts on iGAS. Data were collected in academic referral hospitals, teaching hospitals, and regional hospitals ensuring a wide coverage of clinical presentations and severity. Moreover, contributing hospitals were geographically spread throughout the Netherlands, limiting the effects of potential regional differences. We aimed to reduce the risk of selection bias, by having microbiological samples as the main identifier for eligible cases. By including positive isolates from both sterile and nonsterile compartments, we aimed to include an accurate representation of the variety of clinical manifestations. To limit inclusion bias, all retrospective cases were included and the need for informed consent was waived. Moreover, when it was not possible to obtain informed consent from prospective iGAS cases, these cases could be included with the collection of only concise data, without informed consent.

This study also has limitations. Due to the observational and partial retrospective nature of the study design, not all parameters were available for all patients. Therefore, not all detailed analyses on clinical and laboratory features could be performed for the entire cohort (from 2015 until 2024). There probably was a selection bias for more complicated cases of pulmonary infections by iGAS in our study because only children with positive sputum, blood, or empyema microbiological confirmation of GAS were included. Children with soft-tissue infections, like cellulitis, required a positive GAS culture and/or polymerase chain reaction test to avoid confounding with other pathogens, such as *Staphylococcus aureus*.

A potential bias is the prospective case-finding method, which relied on inclusion by clinical presentation and microbiological database system screening. For retrospective case-finding, microbiological database system screening was the predominant method for patient identification, which may have resulted in an underrepresentation of STSS and NF cases without microbiological confirmation. However, the effect of this underrepresentation is most likely minimal because STSS and NF were extremely evidenced by data from the mandatory notification of these diseases.^39^ Also, in the post–COVID-19 period, all 8 NF cases (100%) had microbiological confirmation, suggesting that underrepresentation in the pre–COVID-19 period is likely infrequent.

Additionally, because bacteriological surveillance for iGAS started in 2019 at sentinel sites and was only extended to national level coverage in April 2022, we only had *emm*-type information available in one-half of the post–COVID-19 pandemic cases. Therefore, we could not make reliable comparisons between pre– and post–COVID-19 *emm*-types.

## Conclusions

In this cohort study we found that involvement of the lungs or central nervous system, STSS, impaired consciousness or pulmonary symptoms, elevated C-reactive protein levels, and reduced estimated glomerular filtration rate were associated with severe pediatric iGAS. From 2022, iGAS incidence and severity rose sharply in the Netherlands after an initial decline during the COVID-19 pandemic, with a marked increase in the incidence of pulmonary infections, NF, meningitis or encephalitis, and STSS. Since the spring of 2024, iGAS incidence has returned to baseline levels, but vigilance remains warranted because pediatric iGAS infections have a high morbidity and mortality. The emergence or reemergence of infectious pathogens should be carefully monitored by clinical reporting systems with sufficient detail and real-time reporting.

## Notes

Supplement 1. **eFigure 1.** Geographical Location of COPP-iGAS Consortium Hospital in the Netherlands **eTable 1.** Sterile Body Compartments **eFigure 2.** iGAS manifestations per patient **eTable 2.** Clinical Characteristics for iGAS Cases With Detailed Data Collection **eTable 3.** Clinical and Prodromal Signs in Children With iGAS **eFigure 3.** Risk Factors for Mortality in Children With iGAS **eFigure 4.** Risk Factors for Severe Disease in Children With Pulmonary iGAS **eFigure 5.** Risk Factors for Severe Disease in Children With Non-Pulmonary iGAS Infection **eFigure 6.** Clinical Risk Factors for Severe Disease in Children With iGAS **eFigure 7.** Estimated Glomerular Filtration Rate and C-Reactive Protein Values at First Presentation **eTable 4.** Method and Location of GAS Detection Per Clinical Syndrome **eTable 5.** Causative *emm*-Type in Pediatric iGAS Patients With Detailed Data Collection **eFigure 8.** Incidence and Clinical Characteristics of iGAS *emm*-Types **eTable 6.** Comparison of *emm*-Types for Disease Severity in Pediatric iGAS Patients **eTable 7.** Multivariate Logistic Regression Model of Causative *emm*-Type on Severity **eTable 8.** *emm*-Types in Different iGAS Clinical Groups **eTable 9.** Incidence Rate Ratio Pre- vs Post-COVID-19 for All iGAS Cases and iGAS Clinical Groups

Supplement 2.
Data Sharing Statement
