# Supplementary material for: Risk Factors for Severe Pediatric Invasive Group A Streptococcal Disease
Source: JAMA Netw Open. 2025 Aug 19;8(8):e2527717. doi: 10.1001/jamanetworkopen.2025.27717 (PMC12365701; doi:10.1001/jamanetworkopen.2025.27717)
Supplement: Supplement 1. — eFigure 1. Geographical Location of COPP-iGAS Consortium Hospital in the Netherlands eTable 1. Sterile Body Compartments eFigure 2. iGAS manifestations per patient eTable 2. Clinical Characteristics for iGAS Cases With Detailed Data Collection eTable 3. Clinical and Prodromal Signs in Children With iGAS eFigure 3. Risk Factors for Mortality in Children With iGAS eFigure 4. Risk Factors for Severe Disease in Children With Pulmonary iGAS eFigure 5. Risk Factors for Severe Disease in Children With Non-Pulmonary iGAS Infection eFigure 6. Clinical Risk Factors for Severe Disease in Children With iGAS eFigure 7. Estimated Glomerular Filtration Rate and C-Reactive Protein Values at First Presentation eTable 4. Method and Location of GAS Detection Per Clinical Syndrome eTable 5. Causative emm-Type in Pediatric iGAS Patients With Detailed Data Collection eFigure 8. Incidence and Clinical Characteristics of iGAS emm-Types eTable 6. Comparison of emm-Types for Disease Severity in Pediatric iGAS Patients eTable 7. Multivariate Logistic Regression Model of Causative emm-Type on Severity eTable 8. emm-Types in Different iGAS Clinical Groups eTable 9. Incidence Rate Ratio Pre- vs Post-COVID-19 for All iGAS Cases and iGAS Clinical Groups [file jamanetwopen-e2527717-s001.pdf]

Supplemental Online Content

van Kempen EB, Tulling AJ, von Asmuth EJ, et al. Risk factors for severe pediatric invasive group A Streptococcal disease. *JAMA Netw Open.* 2025;8(8):e2527717. doi:10.1001/jamanetworkopen.2025.27717

eFigure 1. Geographical Location of COPP-iGAS Consortium Hospital in the Netherlands

eTable 1. Sterile Body Compartments

eFigure 2. iGAS manifestations per patient

eTable 2. Clinical Characteristics for iGAS Cases With Detailed Data Collection

eTable 3. Clinical and Prodromal Signs in Children With iGAS

eFigure 3. Risk Factors for Mortality in Children With iGAS

eFigure 4. Risk Factors for Severe Disease in Children With Pulmonary iGAS

eFigure 5. Risk Factors for Severe Disease in Children With Non-Pulmonary iGAS Infection

eFigure 6. Clinical Risk Factors for Severe Disease in Children With iGAS

eFigure 7. Estimated Glomerular Filtration Rate and C-Reactive Protein Values at First Presentation

eTable 4. Method and Location of GAS Detection Per Clinical Syndrome

eTable 5. Causative *emm*-Type in Pediatric iGAS Patients With Detailed Data Collection

eFigure 8. Incidence and Clinical Characteristics of iGAS *emm*-Types

eTable 6. Comparison of *emm*-Types for Disease Severity in Pediatric iGAS Patients

eTable 7. Multivariate Logistic Regression Model of Causative *emm*-Type on Severity

eTable 8. *emm*-Types in Different iGAS Clinical Groups

eTable 9. Incidence Rate Ratio Pre- vs Post-COVID-19 for All iGAS Cases and iGAS Clinical Groups

This supplemental material has been provided by the authors to give readers additional information about their work.

© 2025 van Kempen EB et al. *JAMA Network Open*.

eFigure 1: COPP-iGAS Consortium Hospitals

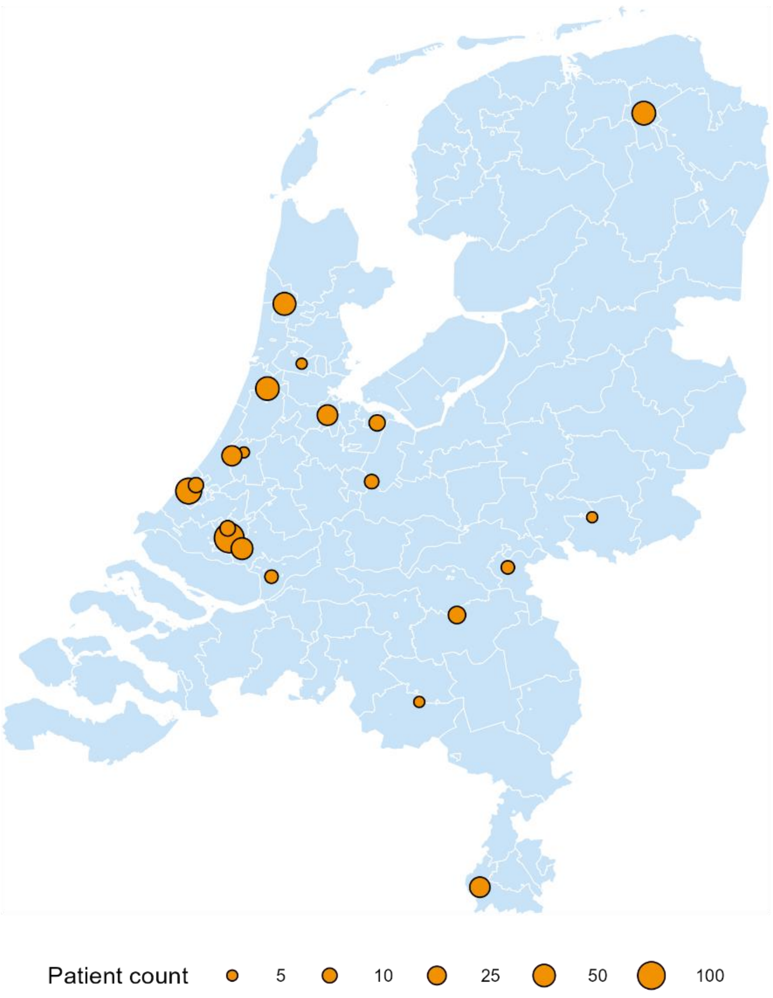

eFigure 1: Geographical location of COPP-iGAS consortium hospitals in the Netherlands.

© 2025 van Kempen EB et al. *JAMA Network Open*.

eTable 1: Overview of Sterile Body Compartments

Normally sterile body site
Blood
Urine
Cerebrospinal fluid (CSF)
Pleural fluid
Peritoneal fluid
Pericardial fluid
Bone (bone and bone marrow)
Joint fluid, includes:<br>• synovial fluid<br>• fluid, needle aspirate, or culture of any specific joint
Internal body sites,<br>• lymph node<br>• brain<br>• heart<br>• liver<br>• spleen<br>• vitreous fluid<br>• kidney<br>• pancreas<br>• ovary<br>• vascular tissue<br>• muscle

eTable 1: Description of all sterile body compartments where GAS could potentially be detected.

© 2025 van Kempen EB et al. *JAMA Network Open*.

eFigure 2: iGAS Manifestations Per Patient

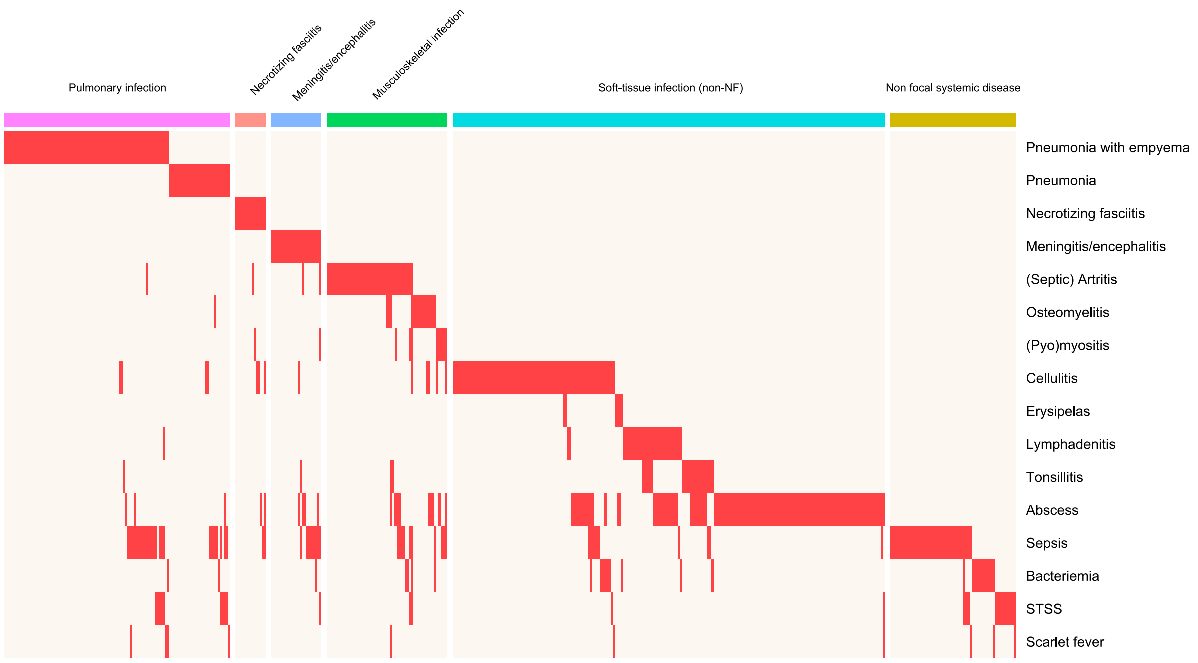

eFigure 2: This map demonstrates that patients often have multiple iGAS manifestations. On the x-axis (columns) 515 cases are plotted. The y-axis (rows) displays the possible iGAS manifestations one can have, each with a yes (in red) and no option. The upper bar shows the main clinical groupings, based on initial focus of iGAS infection. Patients in the “Other” clinical group are not shown (n=102).

© 2025 van Kempen EB et al. *JAMA Network Open*.

eTable 2: Clinical Characteristics for iGAS Cases With Detailed Data Collection

	**Overall** (n=192)	**Pulmonary infection** (n=54)	**Meningitis / encephalitis** (n=12)	**Necrotizing fasciitis** (n=8)	**Musculoskeletal infection** (n=25)	**Soft tissue infection (non-NF)** (n=37)	**Non focal systemic disease** (n=23)	**Other** (n=33)	**p-value**
**Sex (% male)**	91/192 (47)	30/54 (56)	6/12 (50)	2/8 (25)	12/25 (48)	18/37 (49)	9/23 (39)	14/33 (42)	.83
**Age (continuous, years) (IQR)**	4.2 (1.7, 7.2)	3.2 (1.5, 7.2)	5.1 (3.3, 7.0)	3.2 (2.8, 7.6)	6.0 (3.8, 8.0)	2 .9 (1.4, 6.6)	2 .2 (1.7, 6.9)	4.5 (1.6, 6.0)	.57
**Medical history present (%)**	90/192 (47)	21/54 (39)	6/12 (50)	3/8 (38)	12/25 (48)	19/37 (51)	11/23 (48)	18/33 (55)	.91
**ENT history (%)**	27/192 (14)	4/54 (7.4)	4/12 (33)	0/8 (0)	2/25 (8.0)	3/37 (8.1)	3/23 (13)	11/33 (33)	.031
**Pulmonary history (%)**	21/192 (11)	6/54 (11)	0/12 (0)	1/8 (12)	1/25 (4.0)	6/37 (16)	1/23 (4.3)	6/33 (18)	.58
**Cardiac history (%)**	10/192 (5.2)	5/54 (9.3)	1/12 (8.3)	0/8 (0)	1/25 (4.0)	0/37 (0)	1/23 (4.3)	2/33 (6.1)	.70
**GI history (%)**	6/192 (3.1)	2/54 (3.7)	0/12 (0)	0/8 (0)	0/25 (0)	0/37 (0)	0/23 (0)	4/33 (12)	.28
**Neurological history (%)**	6/192 (3.1)	2/54 (3.7)	0/12 (0)	0/8 (0)	1/25 (4.0)	1/37 (2.7)	1/23 (4.3)	1/33 (3.0)	1.0
**Neurodevelopmental problems (%)**	7/192 (3.6)	3/54 (5.6)	0/12 (0)	0/8 (0)	3/25 (12)	0/37 (0)	0/23 (0)	1/33 (3.0)	.46
**Urology/Nephrology history (%)**	5/192 (2.6)	1/54 (1.9)	0/12 (0)	0/8 (0)	1/25 (4.0)	1/37 (2.7)	2/23 (8.7)	0/33 (0)	.71
**Haematological history (%)**	2/192 (1.0)	0/54 (0)	0/12 (0)	0/8 (0)	0/25 (0)	0/37 (0)	2/23 (8.7)	0/33 (0)	.11
**Inborn Errors of Immunity history (%)**	0/192 (0)	0/54 (0)	0/12 (0)	0/8 (0)	0/25 (0)	0/37 (0)	0/23 (0)	0/33 (0)	NA
**Metabolic disease (%)**	3/192 (1.6)	0/54 (0)	0/12 (0)	0/8 (0)	1/25 (4.0)	0/37 (0)	1/23 (4.3)	1/33 (3.0)	.58
**Dermatological history (%)**	15/192 (7.8)	3/54 (5.6)	1/12 (8.3)	0/8 (0)	2/25 (8.0)	5/37 (14)	4/23 (17)	0/33 (0)	.30
**Allergy history (%)**	5/192 (2.6)	1/54 (1.9)	0/12 (0)	0/8 (0)	1/25 (4.0)	1/37 (2.7)	2/23 (8.7)	0/33 (0)	.71
**Endocrinology history (%)**	1/192 (0.5)	0/54 (0)	0/12 (0)	1/8 (12)	0/25 (0)	0/37 (0)	0/23 (0)	0/33 (0)	.010
**History of prematurity (%)**	2/192 (1.0)	2/54 (3.7)	0/12 (0)	0/8 (0)	0/25 (0)	0/37 (0)	0/23 (0)	0/33 (0)	.89
**Oncological history (%)**	0/192 (0)	0/54 (0)	0/12 (0)	0/8 (0)	0/25 (0)	0/37 (0)	0/23 (0)	0/33 (0)	NA
**Rheumatology history (%)**	3/192 (1.6)	1/54 (1.9)	0/12 (0)	0/8 (0)	0/25 (0)	1/37 (2.7)	0/23 (0)	1/33 (3.0)	1.0
**Admission duration (days) (IQR)**	8 (5, 16)	16 (9, 20)	9 (4, 15)	11 (10, 12)	7 (4, 10)	5 (3, 9)	6 (5, 9)	7 (3, 12)	<.001
**ICU admission duration (days) (IQR)**	2 .5 (1.0, 6.0)	2.0 (1.0, 6.0)	1.0 (1.0, 3.5)	4.0 (4.0, 7.0)	4.0 (2.0, 4.0)	3.5 (1.5, 6.2)	2.0 (1.0, 7.0)	1.0 (1.0, 6.0)	.91

eTable 2: Clinical characteristics in children with iGAS disease for cases with detailed data collection. The values are presented by count and non-missing denominator (n/N (%)) or median and interquartile ranges. Patients were allocated to a specific disease category based on a decision making model. For statistical testing the Fisher's Exact test or Kruskal-Wallis rank sum test were performed. Multiple testing correction was performed using the Benjamini-Hochberg procedure. *Abbreviations*: NF: Necrotizing fasciitis, ENT: Ear-nose-throat, GI: Gasto-intestinal, ICU: Intensive Care Unit.

© 2025 van Kempen EB et al. *JAMA Network Open*.

eTable 3: Clinical and Prodromal Signs in Children With iGAS

	**Overall** (n=617)	**Pulmonary infection**, (n=118)	**Meningitis / encephalitis** (n=26)	**Necrotizing fasciitis**, (n=16)	**Musculoskeletal infection**, (n=63)	**Soft tissue infection (non-NF)** (n=226)	**Non focal systemic disease** (n=66)	**Other** (n=102)	**p-value**
**Age (stratified, years) (%)**									.23
0-4 years	351/617 (57)	71/118 (60)	12/26 (46)	11/16 (69)	34/63 (54)	125/226 (55)	42/66 (64)	56/102 (55)	
5-9 years	166/617 (27)	33/118 (28)	7/26 (27)	2/16 (12)	21/63 (33)	54/226 (24)	13/66 (20)	36/102 (35)	
10 years and older	100/617 (16)	14/118 (12)	7/26 (27)	3/16 (19)	8/63 (13)	47/226 (21)	11/66 (17)	10/102 (9.8)	
**COVID-19 pandemic periods (%)**									.0027
01/2015-03/2020	218/617 (35)	28/118 (24)	3/26 (12)	1/16 (6.2)	19/63 (30)	114/226 (50)	18/66 (27)	35/102 (34)	
04/2020-12/2021	12/617 (1.9)	1/118 (0.8)	0/26 (0)	0/16 (0)	4/63 (6.3)	3/226 (1.3)	2/66 (3.0)	2/102 (2.0)	
01/2022-05/2024	387/617 (63)	89/118 (75)	23/26 (88)	15/16 (94)	40/63 (63)	109/226 (48)	46/66 (70)	65/102 (64)	
**Severe Disease (%)**	195/617 (32)	84/118 (71)	17/26 (65)	12/16 (75)	8/63 (13)	18/226 (8.0)	35/66 (53)	21/102 (21)	<.001
**ICU admission (%)**	182/617 (29)	83/118 (70)	15/26 (58)	12/16 (75)	8/63 (13)	17/226 (7.5)	30/66 (45)	17/102 (17)	.0027
**Mortality (%)**	28/617 (4.5)	5/118 (4.2)	4/26 (15)	0/16 (0)	0/63 (0)	1/226 (0.4)	10/66 (15)	8/102 (7.8)	.0027
**Preceding skin or pulmonary signs (%)**	357/592 (60)	76/114 (67)	15/26 (58)	14/16 (88)	35/62 (56)	131/217 (60)	39/65 (60)	47/92 (51)	.21
Preceding skin signs	194/573 (34)	22/108 (20)	7/26 (27)	14/16 (88)	25/61 (41)	89/212 (42)	18/65 (28)	19/85 (22)	<.001
Preceding pulmonary signs	201/572 (35)	65/113 (58)	9/26 (35)	0/15 (0)	18/60 (30)	52/206 (25)	25/62 (40)	32/90 (36)	<.001
Preceding VZV infection	85/570 (15)	9/108 (8.3)	3/25 (12)	12/16 (75)	12/61 (20)	38/211 (18)	6/65 (9.2)	5/84 (6.0)	.0027

eTable 3: Clinical and prodromal signs in children with iGAS. The values are presented by count and non-missing denominator (n/N (%)). Patients were allocated to a specific disease category based on a decision making model. For the comparison of age, period of infection, ICU admission, mortality, and preceding VZV infection a Fisher's Exact test for Count data with simulated p-value (based on 2000 replicates) was performed. For other variables the Chi-squared test was used. Multiple testing correction was performed using the Benjamini-Hochberg procedure. *Abbreviations*: NF: Necrotizing fasciitis, ICU: Intensive Care Unit, VZV: Varicella Zoster Virus.

© 2025 van Kempen EB et al. *JAMA Network Open*.

eFigure 3: Risk Factors for Mortality in Children With iGAS

Characteristic	Odds Ratio	Univariate Analysis	Multivariate Analysis	Severity Within group	Severity Outside group
Age (stratified)					
0–4		Reference		18/351 (5%)	10/266 (4%)
5–9		0.69 (0.27 ; 1.78 P:0.631)		6/166 (4%)	22/451 (5%)
10–17		0.77 (0.25 ; 2.33 P:0.803)		4/100 (4%)	24/517 (5%)
COVID–19 period					
01/2015–03/2020		Reference		3/218 (1%)	24/387 (6%)
01/2022–05/2024		4.74 (1.41 ; 15.92 P:0.0398)		24/387 (6%)	3/218 (1%)
Preceding pulmonary or skin sign/infection		0.87 (0.40 ; 1.88 P:0.848)		16/357 (4%)	12/235 (5%)
Preceding skin sign/infection		0.43 (0.16 ; 1.15 P:0.201)		5/194 (3%)	22/379 (6%)
Preceding airway infection / flu–like symptoms		1.51 (0.69 ; 3.29 P:0.477)		12/201 (6%)	15/371 (4%)
Preceding varicella zoster infection		0.70 (0.21 ; 2.39 P:0.729)		3/85 (4%)	24/485 (5%)
Pulmonary involvement		0.92 (0.34 ; 2.46 P:0.928)		5/118 (4%)	23/499 (5%)
Necrotizing fasciitis		0.61 (0.00 ; 4.72 P:0.843)		0/16 (0%)	28/601 (5%)
Lymphadenitis/Tonsillitis		0.17 (0.00 ; 1.23 P: 0.2)		0/55 (0%)	28/562 (5%)
Dermatological involvement		0.08 (0.00 ; 0.60 P:0.0264)	0.08 (0.00 ; 0.58 P:0.0237)	0/102 (0%)	28/515 (5%)
Abscess present		0.11 (0.02 ; 0.83 P:0.088)	0.16 (0.02 ; 0.64 P:0.0237)	1/147 (1%)	27/470 (6%)
Musculoskeletal infection		0.13 (0.00 ; 0.95 P:0.105)	0.11 (0.00 ; 0.79 P:0.0643)	0/69 (0%)	28/548 (5%)
Meningitis/encephalitis		4.30 (1.37 ; 13.44 P:0.0406)		4/26 (15%)	24/591 (4%)
STSS		6.71 (2.48 ; 18.14 P:0.00113)	4.92 (1.71 ; 12.84 P:0.0195)	6/29 (21%)	22/588 (4%)
Sex (male)		1.11 (0.27 ; 4.59 P:0.942)		4/91 (4%)	4/101 (4%)
Medical history present		0.36 (0.07 ; 1.85 P:0.389)		2/90 (2%)	6/102 (6%)

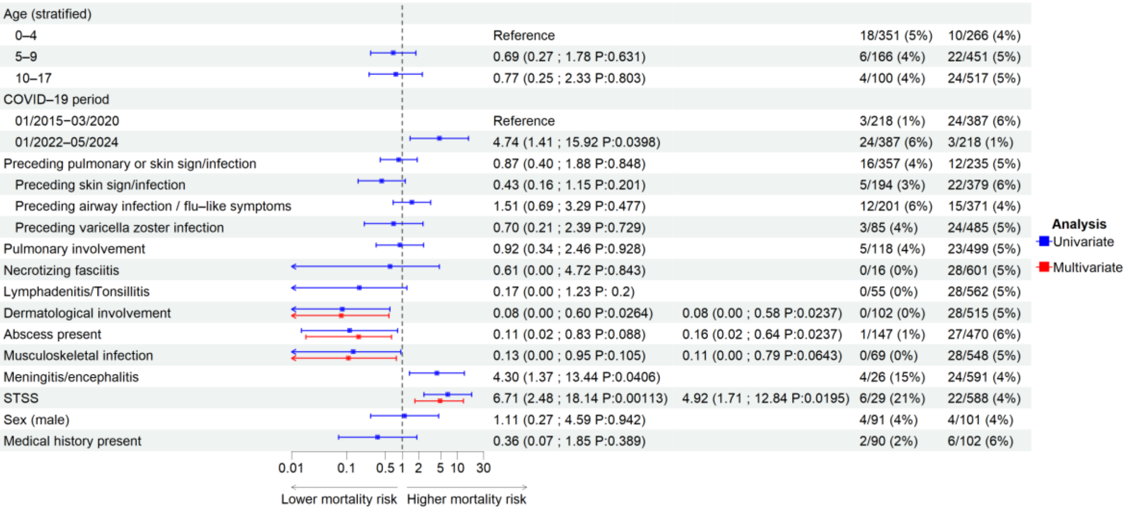

eFigure 3: Risk factors for mortality in children diagnosed with iGAS infection. Odds Ratios, 95% Confidence intervals and p-values (OR (CI95% p-value)) were calculated based on a univariate and multivariate logistic regression model. Variable selection for multivariate analysis was performed using forward and backward selection optimized using the Bayesian information criterion. Data only available for patients with detailed data extraction were not included in the generation of the multivariate model (Sex and prior medical history). The Odds ratios, confidence intervals and p-values for the following variables were calculated using logistic regression with Firth's correction: Dermatological involvement, necrotizing fasciitis, lymphadenitis / tonsilitis, and musculoskeletal infection. Multiple testing correction was performed using the Benjamini-Hochberg procedure.

© 2025 van Kempen EB et al. *JAMA Network Open*.

eFigure 4: Risk Factors for Severe Disease in Children With Pulmonary iGAS (N=118)

Characteristic	Odds Ratio	Univariate Analysis	Multivariate Analysis	Severity Within group	Severity Outside group
Age (stratified)					
0–4		Reference		57/71 (80%)	27/47 (57%)
5–9		0.38 (0.15 ; 0.94 P:0.0944)		20/33 (61%)	64/85 (75%)
10–17		0.25 (0.07 ; 0.82 P:0.0629)		7/14 (50%)	77/104 (74%)
COVID–19 period					
01/2015–03/2020		Reference		19/28 (68%)	64/89 (72%)
01/2022–05/2024		1.21 (0.48 ; 3.04 P:0.825)		64/89 (72%)	19/28 (68%)
Preceding pulmonary or skin sign/infection		0.58 (0.23 ; 1.45 P:0.401)		52/76 (68%)	30/38 (79%)
Preceding skin sign/infection		0.50 (0.19 ; 1.32 P:0.299)		13/22 (59%)	64/86 (74%)
Preceding airway infection / flu–like symptoms		0.97 (0.42 ; 2.24 P:0.995)		47/65 (72%)	35/48 (73%)
Preceding varicella zoster infection		0.79 (0.18 ; 3.37 P:0.865)		6/9 (67%)	71/99 (72%)
Pulmonary involvement				84/118 (71%)	0/0
Necrotizing fasciitis				0/0	84/118 (71%)
Lymphadenitis/Tonsillitis		0.40 (0.02 ; 6.55 P:0.692)		1/2 (50%)	83/116 (72%)
Dermatological involvement		0.12 (0.01 ; 1.24 P:0.173)		1/4 (25%)	83/114 (73%)
Abscess present		2.96 (0.28 ; 402.49 P:0.606)		3/3 (100%)	81/115 (70%)
Musculoskeletal infection		0.40 (0.02 ; 6.55 P:0.692)		1/2 (50%)	83/116 (72%)
Meningitis/encephalitis				0/0	84/118 (71%)
STSS		3.47 (0.42 ; 28.90 P:0.412)		8/9 (89%)	76/109 (70%)
Sex (male)		0.45 (0.13 ; 1.56 P:0.374)		19/30 (63%)	19/24 (79%)
Medical history present		0.36 (0.11 ; 1.19 P:0.203)		12/21 (57%)	26/33 (79%)

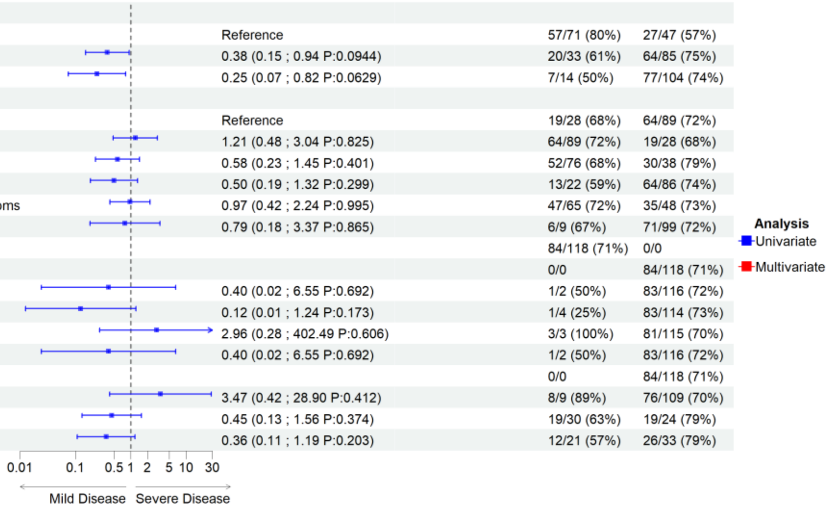

eFigure 4: Risk factors for disease severity (ICU admission and/or mortality) in children diagnosed with iGAS infection with pulmonary involvement. Odds Ratios, 95% Confidence intervals and p-values (OR (CI95% p-value)) were calculated based on a univariate and multivariate logistic regression model. Variable selection for multivariate analysis was performed using forward and backward selection optimized using the Bayesian information criterion. Data only available for patients with detailed data extraction were not included in the generation of the multivariate model (Sex and prior medical history). The Odds ratios, confidence intervals and p-values for the following variables were calculated using logistic regression with Firth's correction: Abscess present. Both Necrotizing fasciitis and meningitis / encephalitis did not concomitantly occur in patients with pulmonary involvement and calculation of odds ratios are thus not feasible. Multiple testing correction was performed using the Benjamini-Hochberg procedure.

© 2025 van Kempen EB et al. *JAMA Network Open*.

eFigure 5: Risk Factors for Severe Disease in Children With Non-Pulmonary iGAS Infection (N=499)

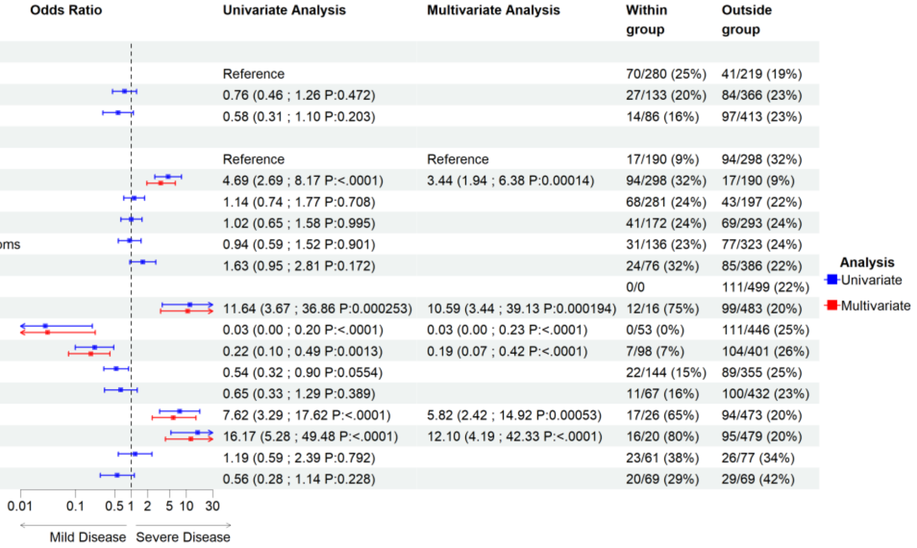

Characteristic	Univariate Analysis	Multivariate Analysis	Severity Within group	Severity Outside group
Age (stratified)				
0–4	Reference		70/280 (25%)	41/219 (19%)
5–9	0.76 (0.46 ; 1.26 P:0.472)		27/133 (20%)	84/366 (23%)
10–17	0.58 (0.31 ; 1.10 P:0.203)		14/86 (16%)	97/413 (23%)
COVID–19 period				
01/2015–03/2020	Reference	Reference	17/190 (9%)	94/298 (32%)
01/2022–05/2024	4.69 (2.69 ; 8.17 P:<.0001)	3.44 (1.94 ; 6.38 P:0.00014)	94/298 (32%)	17/190 (9%)
Preceding pulmonary or skin sign/infection	1.14 (0.74 ; 1.77 P:0.708)		68/281 (24%)	43/197 (22%)
Preceding skin sign/infection	1.02 (0.65 ; 1.58 P:0.995)		41/172 (24%)	69/293 (24%)
Preceding airway infection / flu–like symptoms	0.94 (0.59 ; 1.52 P:0.901)		31/136 (23%)	77/323 (24%)
Preceding varicella zoster infection	1.63 (0.95 ; 2.81 P:0.172)		24/76 (32%)	85/386 (22%)
Pulmonary involvement			0/0	111/499 (22%)
Necrotizing fasciitis	11.64 (3.67 ; 36.86 P:0.000253)	10.59 (3.44 ; 39.13 P:0.000194)	12/16 (75%)	99/483 (20%)
Lymphadenitis/Tonsillitis	0.03 (0.00 ; 0.20 P:<.0001)	0.03 (0.00 ; 0.23 P:<.0001)	0/53 (0%)	111/446 (25%)
Dermatological involvement	0.22 (0.10 ; 0.49 P:0.0013)	0.19 (0.07 ; 0.42 P:<.0001)	7/98 (7%)	104/401 (26%)
Abscess present	0.54 (0.32 ; 0.90 P:0.0554)		22/144 (15%)	89/355 (25%)
Musculoskeletal infection	0.65 (0.33 ; 1.29 P:0.389)		11/67 (16%)	100/432 (23%)
Meningitis/encephalitis	7.62 (3.29 ; 17.62 P:<.0001)	5.82 (2.42 ; 14.92 P:0.00053)	17/26 (65%)	94/473 (20%)
STSS	16.17 (5.28 ; 49.48 P:<.0001)	12.10 (4.19 ; 42.33 P:<.0001)	16/20 (80%)	95/479 (20%)
Sex (male)	1.19 (0.59 ; 2.39 P:0.792)		23/61 (38%)	26/77 (34%)
Medical history present	0.56 (0.28 ; 1.14 P:0.228)		20/69 (29%)	29/69 (42%)

eFigure 5: Risk factors for disease severity (ICU admission and/or mortality) in children diagnosed with non-pulmonary iGAS infection. Odds Ratios, 95% Confidence intervals and p-values (OR (CI95% p-value)) were calculated based on a univariate and multivariate logistic regression model. Variable selection for multivariate analysis was performed using forward and backward selection optimized using the Bayesian information criterion. Data only available for patients with detailed data extraction were not included in the generation of the multivariate model (Sex and prior medical history). The "Lymphadenitis/Tonsilitis" variable was calculated using Firth correction due to complete separation. Multiple testing correction was performed using the Benjamini-Hochberg procedure.

© 2025 van Kempen EB et al. *JAMA Network Open*.

eFigure 6: Clinical Risk Factors for Severe Disease in Children With iGAS (N=192)

Characteristic	Univariate Analysis	Multivariate Analysis	Severity Within group	Severity Outside group
History of ear−nose−throat condition	0.67 (0.29 ; 1.56 P:0.525)	0.68 (0.26 ; 1.81 P:0.63)	10/27 (37%)	77/165 (47%)
History of pulmonary condition	0.72 (0.28 ; 1.82 P:0.665)	0.88 (0.30 ; 2.55 P:0.901)	8/21 (38%)	79/171 (46%)
History of dermatological condition	0.58 (0.19 ; 1.76 P:0.506)	0.41 (0.11 ; 1.51 P:0.325)	5/15 (33%)	82/177 (46%)
Symptom: Fatigue	0.92 (0.50 ; 1.72 P:0.901)	1.00 (0.49 ; 2.02 P: 1)	25/58 (43%)	59/131 (45%)
Symptom: Diarrhea	1.53 (0.70 ; 3.35 P:0.466)	0.86 (0.34 ; 2.19 P:0.865)	16/30 (53%)	68/159 (43%)
Symptom: Dermatological sign	1.11 (0.59 ; 2.09 P:0.865)	0.93 (0.45 ; 1.92 P:0.919)	25/54 (46%)	59/135 (44%)
Symptom: Otalgia	0.54 (0.20 ; 1.50 P:0.401)	0.56 (0.16 ; 1.99 P:0.549)	6/19 (32%)	78/170 (46%)
Symptom: Non−productive cough	1.03 (0.40 ; 2.60 P: 1)	0.52 (0.17 ; 1.55 P:0.401)	9/20 (45%)	75/169 (44%)
Symptom: Athralgia	0.16 (0.03 ; 0.72 P:0.0524)	0.25 (0.05 ; 1.38 P:0.228)	2/16 (12%)	82/173 (47%)
Symptom: reduced consciousness	9.94 (2.83 ; 34.92 P:0.00209)	7.61 (1.84 ; 31.41 P:0.0211)	19/22 (86%)	65/167 (39%)
Symptom: Rhinorroe	1.58 (0.65 ; 3.87 P:0.483)	1.40 (0.51 ; 3.84 P:0.692)	12/22 (55%)	72/167 (43%)
Symptom: Productive cough	3.30 (1.21 ; 9.01 P:0.0591)	1.80 (0.58 ; 5.61 P:0.48)	14/20 (70%)	70/169 (41%)
Symptom: Throatache	0.86 (0.31 ; 2.37 P:0.882)	1.35 (0.41 ; 4.43 P:0.783)	7/17 (41%)	77/172 (45%)
Symptom: Nasea	2.18 (1.16 ; 4.11 P:0.0489)	2.09 (1.00 ; 4.35 P:0.117)	33/57 (58%)	51/132 (39%)
Symptom: Stomacheache	0.73 (0.25 ; 2.10 P:0.721)	0.71 (0.21 ; 2.44 P:0.75)	6/16 (38%)	78/173 (45%)
Symptom: Headache	0.97 (0.35 ; 2.72 P: 1)	1.21 (0.34 ; 4.33 P:0.876)	7/16 (44%)	77/173 (45%)
Physical examination: Increased capillary refill time	2.75 (1.37 ; 5.54 P:0.0199)	1.78 (0.81 ; 3.93 P:0.291)	28/44 (64%)	56/144 (39%)
Physical examination: Dyspnea	11.51 (4.74 ; 27.96 P:<.0001)	9.89 (3.04 ; 32.14 P:0.000914)	36/43 (84%)	42/136 (31%)
Physical examination: Abnormal auscultation	8.33 (3.81 ; 18.20 P:<.0001)	6.32 (2.18 ; 18.32 P:0.00371)	39/49 (80%)	44/138 (32%)
Physical examination: Fever	1.23 (0.48 ; 3.15 P:0.824)	0.78 (0.26 ; 2.29 P:0.803)	76/169 (45%)	8/20 (40%)
Physical examination: Body temperature (C)	0.98 (0.76 ; 1.25 P:0.919)	0.91 (0.68 ; 1.22 P:0.699)	72/173 (42%)	NA
Physical examination: Heart rate (z−score)	1.77 (1.25 ; 2.50 P:0.00579)	1.55 (1.04 ; 2.29 P:0.0802)	75/174 (43%)	NA
Physical examination: Breathing rate (z−score)	1.81 (1.36 ; 2.42 P:0.000366)	1.43 (1.02 ; 2.01 P:0.102)	72/162 (44%)	NA
Physical examination: Saturation (%)	0.40 (0.23 ; 0.71 P:0.00802)	0.52 (0.30 ; 0.91 P:0.0621)	73/164 (45%)	NA
Physical examination: Systolic blood pressure (z−score)	0.86 (0.69 ; 1.07 P:0.316)	0.90 (0.69 ; 1.18 P:0.631)	47/110 (43%)	NA
Physical examination: Diastolic blood pressure (z−score)	0.83 (0.64 ; 1.06 P:0.262)	0.95 (0.71 ; 1.27 P:0.848)	47/110 (43%)	NA
Laboratory: Hemoglobin (mmol/L) (per 5)	1.35 (1.02 ; 1.80 P:0.099)	1.48 (1.02 ; 2.15 P:0.103)	72/163 (44%)	NA
Laboratory: Leukocyte count (*10^9/L) (log1p)	0.40 (0.23 ; 0.70 P:0.00574)	0.48 (0.26 ; 0.91 P:0.068)	73/167 (44%)	NA
Laboratory: Lymphocyte count (*10^9/L) (log1p)	0.44 (0.20 ; 0.95 P:0.0944)	0.50 (0.19 ; 1.33 P:0.306)	34/86 (40%)	NA
Laboratory: Neutrophil count (*10^9/L) (log1p)	0.31 (0.12 ; 0.76 P:0.0362)	0.38 (0.12 ; 1.14 P:0.185)	23/72 (32%)	NA
Laboratory: Monocyte count (*10^9/L) (log1p)	0.44 (0.15 ; 1.30 P:0.266)	0.77 (0.23 ; 2.62 P:0.824)	34/84 (40%)	NA
Laboratory: Hematrocrit (%) (per 5)	1.37 (0.90 ; 2.08 P:0.277)	1.41 (0.81 ; 2.47 P:0.389)	38/109 (35%)	NA
Laboratory: Thrombocyte count (*10^9/L) (per 100)	0.77 (0.62 ; 0.97 P:0.0719)	0.78 (0.62 ; 0.98 P:0.0944)	69/151 (46%)	NA
Laboratory: ALAT (U/L) (log1p)	1.44 (0.86 ; 2.39 P:0.302)	1.38 (0.77 ; 2.46 P:0.463)	25/53 (47%)	NA
Laboratory: ASAT (U/L) (log1p)	2.79 (1.31 ; 5.95 P:0.0298)	2.55 (1.11 ; 5.85 P:0.0745)	24/50 (48%)	NA
Laboratory: Glucose (mmol/L)	0.86 (0.65 ; 1.14 P:0.474)	0.78 (0.54 ; 1.12 P:0.316)	35/55 (64%)	NA
Laboratory: Urea (mmol/L) (log1p)	2.36 (1.01 ; 5.49 P:0.111)	3.51 (1.24 ; 9.96 P:0.0554)	43/79 (54%)	NA
Laboratory: eGFR (mL/min/1,73m2) (per 20)	0.75 (0.61 ; 0.93 P:0.027)	0.64 (0.49 ; 0.84 P:0.00693)	33/66 (50%)	NA
Laboratory: Sodium (mmol/L) (per 5)	0.71 (0.45 ; 1.14 P:0.294)	0.65 (0.38 ; 1.12 P:0.245)	54/94 (57%)	NA
Laboratory: Potassium (mmol/L)	1.82 (0.96 ; 3.44 P:0.15)	1.83 (0.87 ; 3.83 P:0.228)	52/92 (57%)	NA
Laboratory: C−reactive protein (mg/L) (per 100)	1.93 (1.39 ; 2.67 P:0.000665)	1.62 (1.10 ; 2.39 P:0.0457)	70/161 (43%)	NA

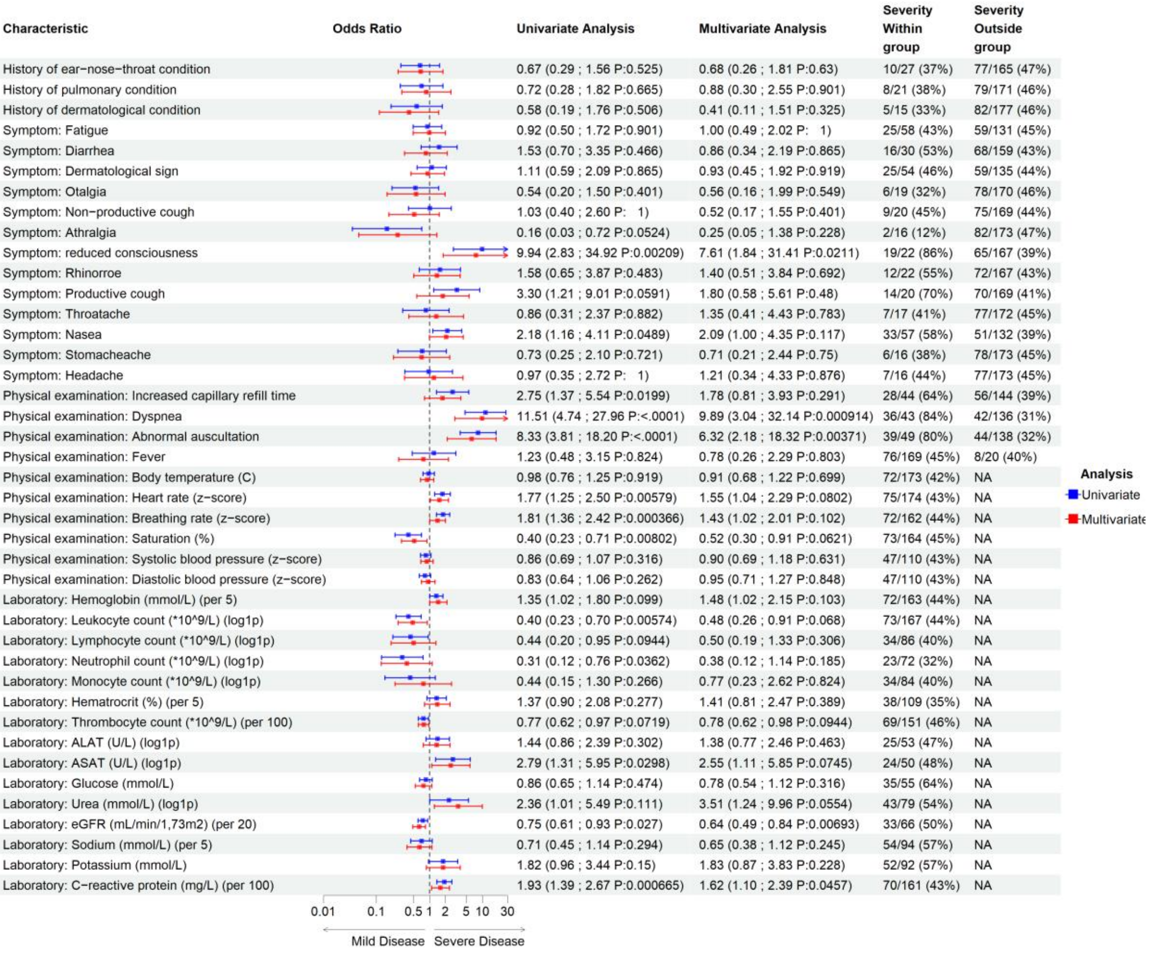

eFigure 6: Clinical risk factors for disease severity (ICU admission and/or mortality) in children diagnosed with iGAS infection. Only variables collected for patients with detailed data extraction are shown. We analysed variables that were positive in at least 15 cases for dichotomous variables and present in at least 50 cases for continuous variables. Odds Ratios were calculated based on a univariate and multivariate logistic regression model. The multivariate model includes the variable of interest adjusted for age, sex, and clinical syndrome. Multiple testing correction was performed using the Benjamini-Hochberg procedure.

© 2025 van Kempen EB et al. *JAMA Network Open*.

eFigure 7: Estimated Glomerular Filtration Rate and C-Reactive Protein Values at First Presentation

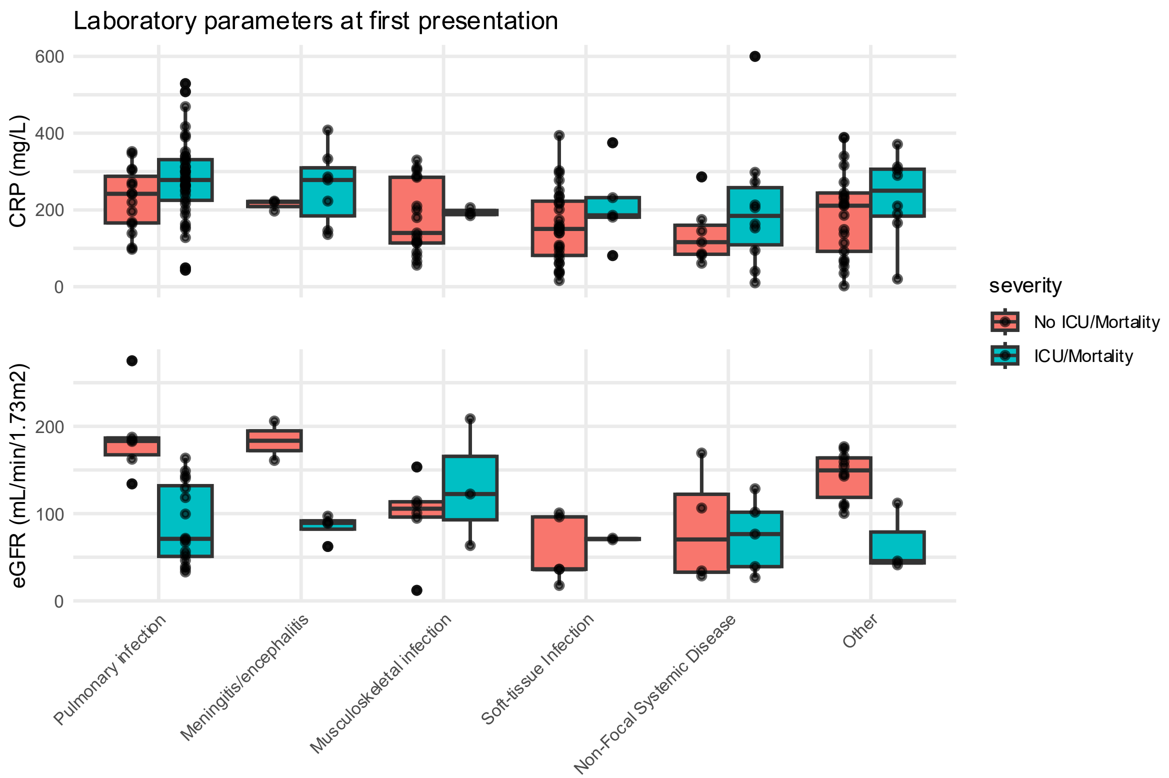

eFigure 7: Estimated Glomerular filtration rate (eGFR) and C-reactive protein (CRP) values at first presentation for different clinical groups and levels of severity. The eGFR was calculated using the modified Schwartz formula.

© 2025 van Kempen EB et al. *JAMA Network Open*.

eTable 4: Method and Location of GAS Detection Per Clinical Syndrome

**Characteristic**	**Overall**, N = 192[1]	**Meningitis/encephalitis**, N = 12[1]	**Musculoskeletal infection**, N = 25[1]	**Necrotizing fasciitis**, N = 8[1]	**Non focal systemic disease**, N = 23[1]	**Pulmonary infection**, N = 54[1]	**Soft tissue infection (Non NF)**, N = 37[1]	**Other**, N = 33[1]
**Body Compartment / circumstance of GAS detection (%)**								
Nasale swab	2 (1·1)	0 (0)	0 (0)	0 (0)	1 (5·0)	0 (0)	0 (0)	1 (3·3)
Pleural fluid	39 (22)	0 (0)	0 (0)	0 (0)	2 (10)	35 (69)	0 (0)	2 (6·7)
Abscess	20 (11)	1 (9·1)	1 (4·3)	1 (12)	1 (5·0)	0 (0)	15 (44)	1 (3·3)
Throat swab	10 (5·6)	1 (9·1)	0 (0)	0 (0)	2 (10)	2 (3·9)	3 (8·8)	2 (6·7)
Nasopharynx swab	2 (1·1)	0 (0)	0 (0)	0 (0)	0 (0)	0 (0)	1 (2·9)	1 (3·3)
Blood	69 (39)	7 (64)	13 (57)	0 (0)	14 (70)	14 (27)	7 (21)	14 (47)
Skin/wound	18 (10)	1 (9·1)	3 (13)	4 (50)	0 (0)	0 (0)	8 (24)	2 (6·7)
Synovial fluid	8 (4·5)	1 (9·1)	7 (30)	0 (0)	0 (0)	0 (0)	0 (0)	0 (0)
Ear	7 (4·0)	2 (18)	0 (0)	0 (0)	0 (0)	0 (0)	0 (0)	5 (17)
Urine	3 (1·7)	0 (0)	0 (0)	0 (0)	1 (5·0)	0 (0)	1 (2·9)	1 (3·3)
Liquor	5 (2·8)	3 (27)	0 (0)	0 (0)	1 (5·0)	0 (0)	0 (0)	1 (3·3)
Sputum	2 (1·1)	0 (0)	0 (0)	0 (0)	1 (5·0)	1 (2·0)	0 (0)	0 (0)
Perioperative	3 (1·7)	0 (0)	0 (0)	0 (0)	0 (0)	0 (0)	0 (0)	3 (10)
Other	22 (12)	1 (9·1)	3 (13)	5 (62)	2 (10)	4 (7·8)	3 (8·8)	4 (13)
Missing microbiology rapport	15 (7·8)	1 (8·3)	2 (8·0)	0 (0)	3 (13)	3 (5·6)	3 (8·1)	3 (9·1)
**Method of GAS detection (%)**								
Culture	160 (83)	10 (83)	23 (92)	6 (75)	19 (83)	42 (78)	32 (86)	28 (85)
PCR and culture	2 (1·0)	1 (8·3)	0 (0)	1 (12)	0 (0)	0 (0)	0 (0)	0 (0)
PCR detection	14 (7·3)	0 (0)	0 (0)	1 (12)	1 (4·3)	9 (17)	2 (5·4)	1 (3·0)
Missing	16 (8·3)	1 (8·3)	2 (8·0)	0 (0)	3 (13)	3 (5·6)	3 (8·1)	4 (12)

eTable 4: Method of GAS detection for the different clinical manifestations. *Abbreviations*: GAS: Group A Streptococcus, PCR: Polymerase chain reaction.

© 2025 van Kempen EB et al. *JAMA Network Open*.

eTable 5: Causative *emm*-Type in Pediatric iGAS Patients With Detailed Data Collection

	Overall (n=101)	2022 (n=32)	2023 (n=45)	2024 (n=24)	p-value
**3.93 Cluster A-C5 (%)**	20 (20)	0 (0)	3 (6.7)	17 (71)	<.0001
**1.0 Cluster A-C3 (%)**	41 (41)	17 (53)	22 (49)	2 (8.3)	.0054[a]
**22.0 Cluster E4 (%)**	9 (8.9)	4 (12)	4 (8.9)	1 (4.2)	.71
**87.0 Cluster E3 (%)**	3 (3.0)	1 (3.1)	2 (4.4)	0 (0)	.89
**12.0 Cluster A-C4 (%)**	7 (6.9)	6 (19)	1 (2.2)	0 (0)	.023
**4.0 Cluster E1 (%)**	11 (11)	3 (9.4)	7 (16)	1 (4.2)	.50
**103.0 Cluster E3 (%)**	1 (1.0)	0 (0)	1 (2.2)	0 (0)	1.0
**1.134 Cluster A-C3 (%)**	3 (3.0)	0 (0)	3 (6.7)	0 (0)	.50
**89.0 Cluster E4 (%)**	1 (1.0)	0 (0)	0 (0)	1 (4.2)	.40
**75.0 Cluster E6 (%)**	1 (1.0)	0 (0)	0 (0)	1 (4.2)	.40
**12.37 Cluster A-C4 (%)**	2 (2.0)	1 (3.1)	0 (0)	1 (4.2)	.48
**156.2 (%)**	1 (1.0)	0 (0)	1 (2.2)	0 (0)	1.0
**1.152 Cluster A-C3 (%)**	1 (1.0)	0 (0)	1 (2.2)	0 (0)	1.0

eTable 5: Specific *emm*-type causing iGAS disease in pediatric patients with detailed data collection from 01/2022 up till 05/2024 for the consecutive years. The Fisher's Exact test and Chi-squared test ([a]) were used with Benjamini-Hochberg correction.

© 2025 van Kempen EB et al. *JAMA Network Open*.

eFigure 8: Incidence and Clinical Characteristics of iGAS *emm*-Types

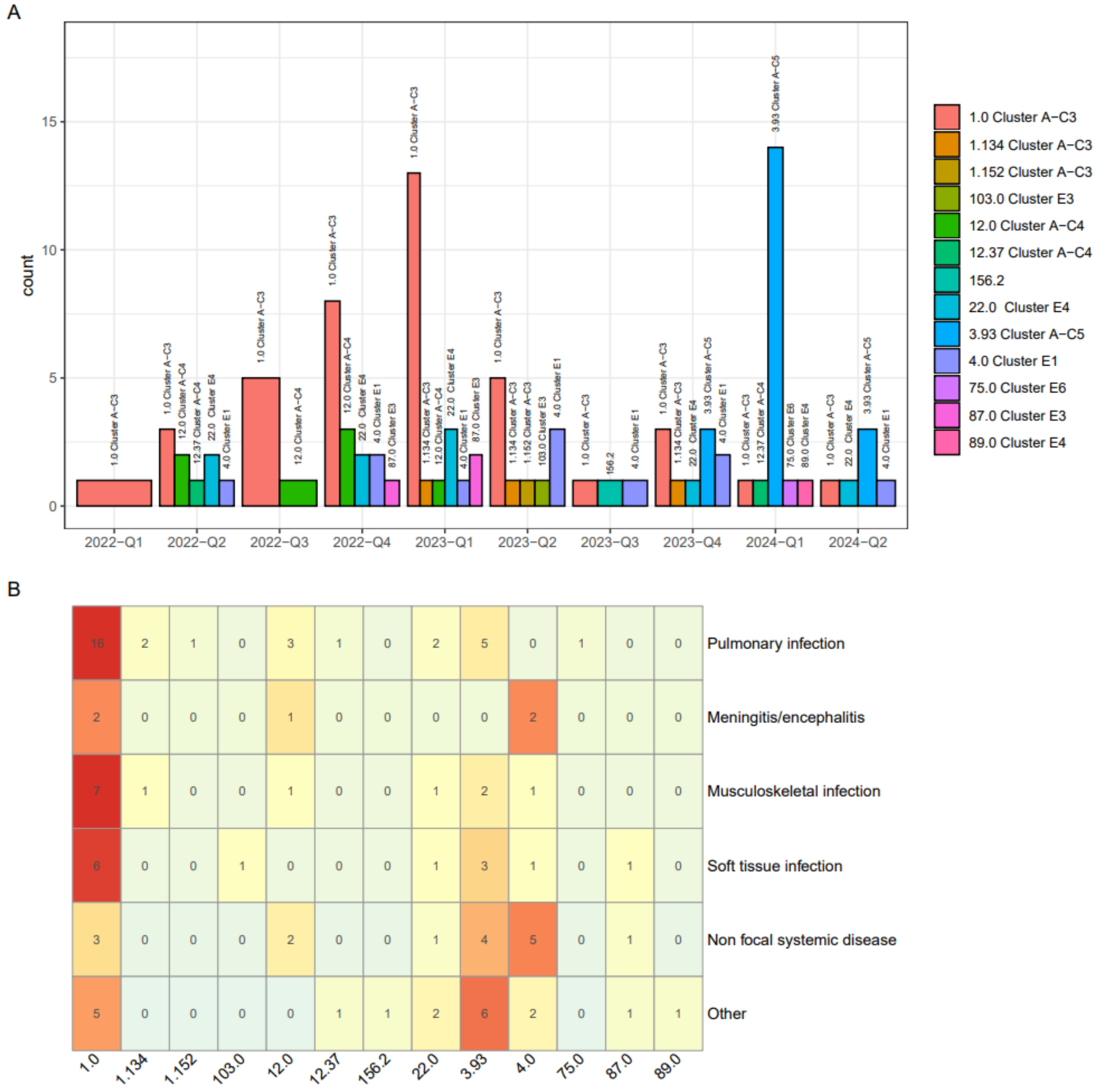

eFigure 8: Incidence and clinical characteristics of different iGAS *emm*-types. **A**: Incidence rate per year and quarter. **B**: Heatmap of different clinical syndromes (rows) by the frequency of a specific causative *emm*-type (columns). Colors were scaled per clinical syndrome.

© 2025 van Kempen EB et al. *JAMA Network Open*.

eTable 6: Comparison of *emm*-Types for Disease Severity in Pediatric iGAS Patients

	ICU/Mortality (n=52)	No ICU/Mortality (n=49)	p-value
**3.93 Cluster A-C5 (%)**	10 (19)	10 (20)	.94[a]
**1.0 Cluster A-C3 (%)**	25 (48)	16 (33)	.23[a]
**22.0 Cluster E4 (%)**	3 (5.8)	6 (12)	.48
**87.0 Cluster E3 (%)**	0 (0)	3 (6.1)	.23
**12.0 Cluster A-C4 (%)**	3 (5.8)	4 (8.2)	.84
**4.0 Cluster E1 (%)**	8 (15)	3 (6.1)	.27[a]
**103.0 Cluster E3 (%)**	0 (0)	1 (2.0)	.67
**1.134 Cluster A-C3 (%)**	2 (3.8)	1 (2.0)	1.0
**89.0 Cluster E4 (%)**	0 (0)	1 (2.0)	.67
**75.0 Cluster E6 (%)**	1 (1.9)	0 (0)	1.0
**12.37 Cluster A-C4 (%)**	0 (0)	2 (4.1)	.40
**156.2 (%)**	0 (0)	1 (2.0)	.67
**1.152 Cluster A-C3 (%)**	0 (0)	1 (2.0)	.67

eTable 6: Specific *emm*-type causing iGAS disease in pediatric patients with detailed data collection from 01/2022 up till 05/2024 for different levels of disease severity. The Fisher's Exact test and Chi-squared test ([a]) were used with Benjamini-Hochberg correction. *Abbreviations*: ICU: Intensive Care Unit.

© 2025 van Kempen EB et al. *JAMA Network Open*.

eTable 7: Multivariate Logistic Regression Model of Causative *emm*-Type on Severity

	Severe disease / cases with *emm*-type (%)	aOR	95% CI	p-value
***Emm*-type**				
Other *Emm*-types	6/20 (30)	—	—	
1.0 Cluster A-C3	25/41 (61)	11.8	2.78, 62.2	.0076
3.93 Cluster A-C5	10/20 (50)	6.06	1.19, 36.1	.094
4.0 Cluster E1	8/11 (73)	13.6	1.88, 127	.044
22.0 Cluster E4	3/9 (33)	2.73	0.34, 22.0	.51

eTable 7: Multivariate logistic regression model for iGAS cases with available data on causative *emm*-type. The model included both *emm*-type, age, sex, and clinical disease group of which only the *emm*-type odds ratio and confidence intervals is shown. All *emm*-types occurring less than 9 times were used as the reference. Multiple testing correction was performed using the Benjamini-Hochberg procedure. *Abbreviations*: aOR: adjusted Odds Ratio, CI: Confidence Interval

eTable 8: *emm*-Types in Different iGAS Clinical Groups

	Pulmonary infection (n=31)	Meningitis/ encephalitis (n=5)	Necrotizing fasciitis (n=4)	Musculoskeletal infection (n=13)	Soft tissue infection (Non NF) (n=13)	Non focal systemic disease (n=16)	Other (n=19)	p-value
**3.93 Cluster A-C5 (%)**	5 (16)	0 (0)	0 (0)	2 (15)	3 (23)	4 (25)	6 (32)	.85
**1.0 Cluster A-C3 (%)**	16 (52)	2 (40)	2 (50)	7 (54)	6 (46)	3 (19)	5 (26)	.40
**22.0 Cluster E4 (%)**	2 (6.5)	0 (0)	2 (50)	1 (7.7)	1 (7.7)	1 (6.2)	2 (11)	.48
**87.0 Cluster E3 (%)**	0 (0)	0 (0)	0 (0)	0 (0)	1 (7.7)	1 (6.2)	1 (5.3)	.69
**12.0 Cluster A-C4 (%)**	3 (9.7)	1 (20)	0 (0)	1 (7.7)	0 (0)	2 (12)	0 (0)	.62
**4.0 Cluster E1 (%)**	0 (0)	2 (40)	0 (0)	1 (7.7)	1 (7.7)	5 (31)	2 (11)	.031
**103.0 Cluster E3 (%)**	0 (0)	0 (0)	0 (0)	0 (0)	1 (7.7)	0 (0)	0 (0)	.52
**1.134 Cluster A-C3 (%)**	2 (6.5)	0 (0)	0 (0)	1 (7.7)	0 (0)	0 (0)	0 (0)	.82
**89.0 Cluster E4 (%)**	0 (0)	0 (0)	0 (0)	0 (0)	0 (0)	0 (0)	1 (5.3)	.83
**75.0 Cluster E6 (%)**	1 (3.2)	0 (0)	0 (0)	0 (0)	0 (0)	0 (0)	0 (0)	1.0
**12.37 Cluster A-C4 (%)**	1 (3.2)	0 (0)	0 (0)	0 (0)	0 (0)	0 (0)	1 (5.3)	1.0
**156.2 (%)**	0 (0)	0 (0)	0 (0)	0 (0)	0 (0)	0 (0)	1 (5.3)	.83
**1.152 Cluster A-C3 (%)**	1 (3.2)	0 (0)	0 (0)	0 (0)	0 (0)	0 (0)	0 (0)	1.0

eTable 8: Specific *emm*-type causing iGAS disease in pediatric patients with detailed data collection from 01/2022 up till 05/2024 for all clinical iGAS groups. The Fisher's Exact test was used with Benjamini-Hochberg correction. *Abbreviations*: NF: Necrotizing fasciitis.

© 2025 van Kempen EB et al. *JAMA Network Open*.

eTable 9: Incidence Rate Ratio Pre- Versus Post-COVID-19 for All iGAS Cases and iGAS Clinical Groups

	**Incidence rate ratio pre-COVID-19 period versus post-COVID-19 period (95% CI)**
**All iGAS cases**	2.93 (2.46;3.49)
**Clinical groups (1 option per patient)**	
Pulmonary infection	5.04 (3.27;7.97)
Meningitis/encephalitis	12.3 (4.14;52.7)
Necrotizing fasciitis	26.1 (5.14; 475)
Musculoskeletal infection	2.74 (1.51;5.07)
Soft tissue infection (non-NF)	1.77 (1.35;2.33)
Non focal systemic disease	4.71 (2.73;8.42)
Other	2.73 (1.76;4.28)
**Clinical involvement (yes/no)**	
Pulmonary involvement	5.04 (3.27;7.97)
Necrotizing fasciitis	26.1 (5.14; 475)
STSS	10.3 (3.88;35.6)
Lymphadenitis/Tonsillitis	1.55 (0.863;2.74)
Dermatological involvement	3.20 (2.10;4.92)
Abscess present	1.56 (1.10;2.19)
Musculoskeletal infection	3.20 (1.80;5.82)
Meningitis/encephalitis	12.3 (4.14;52.7)

eTable 9: The incidence rate ratio of all iGAS cases and iGAS clinical groups between the periods before and after the COVID-19 pandemic were calculated with the patient count per month corrected for different duration of periods using a Poisson regression model. Only patients from hospitals including patients retro- as well as prospectively were included. *Abbreviations*: NF: Necrotizing fasciitis, STSS: Streptococcal toxic shock syndrome

© 2025 van Kempen EB et al. *JAMA Network Open*.

© 2025 van Kempen EB et al. *JAMA Network Open*.
