# Supplementary material for: Risk Factors for Severe Pediatric Invasive Group A Streptococcal Disease
Source: JAMA Netw Open. 2025 Aug 19;8(8):e2527717. doi: 10.1001/jamanetworkopen.2025.27717 (PMC12365701; doi:10.1001/jamanetworkopen.2025.27717)
Supplement: Supplement 2. — Data Sharing Statement [file jamanetwopen-e2527717-s002.pdf]

Data Sharing Statement

van Kempen. Risk Factors for Severe Pediatric Invasive Group A Streptococcal Disease. *JAMA Netw Open*. Published August 19, 2025. doi:10.1001/jamanetworkopen.2025.27717

Data
Data available: Yes
Data types: Deidentified participant data, Data dictionary
How to access data: Individual participant data will be available upon reasonable request, i.e. individual participant data that underlie the results reported in this article, after de-identification. Study protocol is shared on the website www.infectiekids.nl. Data will be available beginning 9 months and ending 36 months following article publication, for researchers who provide a methodologically sound proposal that has been approved by a medical ethical research committee, with analyses that fit the aims in the approved proposal. Proposals may be submitted up to 36 months following article publication. After 36 months the data will be available in our university's data warehouse but without investigator support other than deposited metadata. Information regarding submitting proposals and accessing data may be found at www.infectiekids.nl. Data requestors will need to sign a data access agreement.
When available: beginning date: 01-01-2026, end date: 01-01-2028

Supporting Documents
Document types: Informed consent form
How to access documents: https://infectiekids.nl/voor-professionals/
When available: With publication

Additional Information
Who can access the data: Individual participant data will be available upon reasonable request, i.e. individual participant data that underlie the results reported in this article, after de-identification. Study protocol is shared on the website www.infectiekids.nl. Data will be available beginning 9 months and ending 36 months following article publication, for researchers who provide a methodologically sound proposal that has been approved by a medical ethical research committee, with analyses that fit the aims in the approved proposal. Proposals may be submitted up to 36 months following article publication. After 36 months the data will be available in our university's data warehouse but without investigator support other than deposited metadata. Information regarding submitting proposals and accessing data may be found at www.infectiekids.nl. Data requestors will need to sign a data access agreement.
Types of analyses: Analyses that fit the aims in the approved proposal
Mechanisms of data availability: Individual participant data will be available upon reasonable request, i.e. individual participant data that underlie the results reported in this article, after de-identification. Study protocol is shared on the website www.infectiekids.nl. Data will be available beginning 9 months and ending 36 months following article publication, for researchers who provide a methodologically sound proposal that has been approved by a medical ethical research committee, with analyses that fit the aims in the approved proposal. Proposals may be submitted up to 36 months following article publication. After 36 months the data will be available in our university's data warehouse but without investigator support other than deposited metadata. Information regarding submitting proposals and accessing data may be found at www.infectiekids.nl. Data requestors will need to sign a data access agreement.
